# Measurement and State-Dependent Modulation of Hypoglossal Motor Excitability and Responsivity *In-Vivo*

**DOI:** 10.1038/s41598-019-57328-4

**Published:** 2020-01-17

**Authors:** Jasmin A. Aggarwal, Wen-Ying Liu, Gaspard Montandon, Hattie Liu, Stuart W. Hughes, Richard L. Horner

**Affiliations:** 1 Department of Physiology, University of Toronto, Toronto, M5S 1A8 Canada; 2 State Key Laboratory of Medical Neurobiology and MOE Frontiers Center for Brain Science, Institutes of Brain Science; Department of Pharmacology, School of Basic Medical Science, Fudan University, Shanghai, 200032 China; 3 Institute of Medical Science, University of Toronto, Toronto, M5S 1A8 Canada; 4 Department of Medicine, University of Toronto, Toronto, M5S 1A8 Canada; 5 Keenan Research Centre for Biomedical Science, St. Michael’s Hospital, Toronto, M5B 1W8 Canada; 6 Vertex Pharmaceuticals, Milton, Abingdon OX14 4RW UK

**Keywords:** Motor neuron, Respiration

## Abstract

Motoneurons are the final output pathway for the brain’s influence on behavior. Here we identify properties of hypoglossal motor output to the tongue musculature. Tongue motor control is critical to the pathogenesis of obstructive sleep apnea, a common and serious sleep-related breathing disorder. Studies were performed on mice expressing a light sensitive cation channel exclusively on cholinergic neurons (ChAT-ChR2(H134R)-EYFP). Discrete photostimulations under isoflurane-induced anesthesia from an optical probe positioned above the medullary surface and hypoglossal motor nucleus elicited discrete increases in tongue motor output, with the magnitude of responses dependent on stimulation power (P < 0.001, n = 7) and frequency (P = 0.002, n = 8, with responses to 10 Hz stimulation greater than for 15–25 Hz, P < 0.022). Stimulations during REM sleep elicited significantly reduced responses at powers 3–20 mW compared to non-rapid eye movement (non-REM) sleep and wakefulness (each P < 0.05, n = 7). Response thresholds were also greater in REM sleep (10 mW) compared to non-REM and waking (3 to 5 mW, P < 0.05), and the slopes of the regressions between input photostimulation powers and output motor responses were specifically reduced in REM sleep (P < 0.001). This study identifies that variations in photostimulation input produce tunable changes in hypoglossal motor output *in-vivo* and identifies REM sleep specific suppression of net motor excitability and responsivity.

## Introduction

Obstructive sleep apnea (OSA) is a common and serious breathing disorder with significant clinical, social and economic consequences^1^. OSA is characterized by repeated episodes of upper airway obstruction that occur only during sleep, with the airway obstructions leading to futile breathing efforts, asphyxia, sleep disturbance and other physiological sequalae of medical relevance^2,3^. The root cause of the upper airway obstruction is reduced activity and reflex compensatory responses of the pharyngeal muscles during sleep, whereas in wakefulness the motor activity and responsivity are sufficient to keep the airspace open^4^. Ultimately, state-dependent pharyngeal muscle activity and responsivity of the brainstem motor pools driving these muscles are pivotal to OSA pathophysiology and the phenotypic traits of OSA patients^5–8^.

Such state-dependent activity of the pharyngeal musculature is central to OSA pathogenesis, and the hypoglossal motoneuron pool is the source of motor output to the largest of the pharyngeal muscles, the tongue^2,4^. Single unit recordings of tongue motor activity are an experimentally powerful indicator of the discharge patterns and recruitment characteristics of single hypoglossal motoneurons in the medulla that are otherwise inaccessible for investigation^9–19^. One limitation of such single-unit studies, however, is the inability to impose acute, precise and direct control over hypoglossal motor output to interrogate physiological properties relevant to tongue motor control.

In the present study we use optical stimulation for the acute and temporally-precise control of genetically engineered cells^20^ to modulate hypoglossal motor output and determine changes in state-dependent motor excitability and responsivity from the evoked electromyogram (EMG) responses as measured from recording electrodes in the tongue. The present study tests the hypotheses that: (i) variations in photostimulation input frequency and power delivered from an optical probe positioned above the medullary surface and hypoglossal motor nucleus produce tunable changes in motor output, and (ii) there are specific changes in motor excitability and responsivity occurring across states of wakefulness, non-rapid eye movement (non-REM) sleep and REM sleep. Here we test these hypotheses in ChAT-ChR2(H134R)-EYFP mice expressing a light sensitive cation channel (channelrhodopsin-2, ChR2) exclusively on cholinergic neurons.

The present experiments provide new knowledge of the stimulus-response properties of hypoglossal motor output in an accessible and reproducible preparation *in-vivo* under conditions of isoflurane-induced general anesthesia (Study 1) and across sleep-wake states (Study 2). Moreover, the identified properties are relevant to understanding and manipulating tongue motor control, in particular hypoglossal motor excitability and responsivity, that are key pathophysiological and phenotypic traits related to human OSA^5–8^. Such key properties of hypoglossal motor excitability and responsivity are not are amenable to systematic quantification using previous ‘chemogenetic’ approaches^21,22^ given the inability to impose acute, precise and direct control over hypoglossal motor output using an intervention that can be transiently turned on and off, and graded in intensity, as is possible with photostimulation.

The rationale for Study 1 in anesthetized mice was to interrogate the properties of hypoglossal motor output from the evoked motor responses using different protocols under stable conditions throughout the experiment (i.e., in the absence of spontaneous behaviors). The results from that study then led to the selection of one protocol to address properties of hypoglossal motor excitability and responsivity across naturally occurring sleep-wake states. As such, Study 1 was the necessary prerequisite to Study 2. The presence of phasic inspiratory-modulation during isoflurane anesthesia in Study 1 also allowed the additional investigation of the effects of the optical stimulation in the different protocols on the expression of inspiratory motor activation in the absence of tonic activity.

## Results

### Expression of ChR2(H134R) at the hypoglossal motoneuron pool

**Figure 1:**
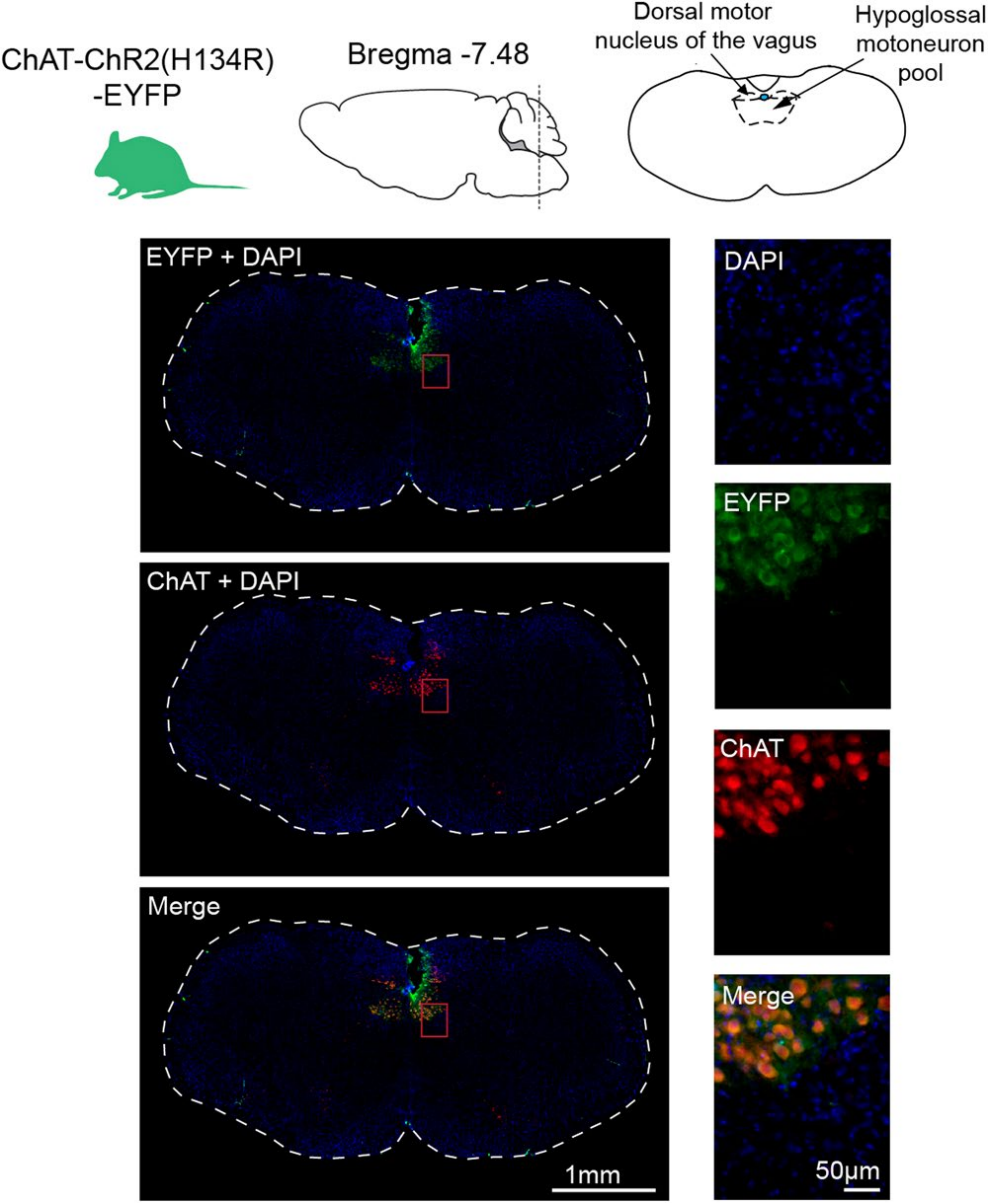
Expression of ChR2(H134R) at the hypoglossal motoneuron pool. Fluorescent microscopy images illustrating the expression of ChR2(H134R)-EYFP and ChAT in coronal brain sections. Shown are low-magnification (left column) and high-magnification (right column) representative images taken from a ChAT-ChR2(H134R)-EYFP mouse at 7.4 mm posterior to bregma stained for DAPI, EYFP and ChAT. The colocalization of EYFP and ChAT at the hypoglossal motoneuron pool is also illustrated (‘merge’).

To confirm the expression of the ChR2(H134R) in hypoglossal motoneurons, the co-expression of enhanced yellow fluorescent protein (EYFP) and choline acetyltransferase (ChAT) was visualized using florescent microscopy. Figure 1 shows representative images of a coronal brain section from one ChAT-ChR2(H134R)-EYFP mouse used in the experiments. The images show colocalization of the ChR2(H134R)-EYFP fusion protein with ChAT in the hypoglossal motoneuron pool.

Cell counts identified that 99.7% of visualized cells that expressed EYFP in the hypoglossal motoneuron pool also expressed ChAT (312 of 313 cells identified from three slices each from three ChAT-ChR2(H134R)-EYFP mice, averaging 7.35 mm posterior to bregma, range 7.1 to 7.5 mm). This result shows that the ChR2(H134R)-EYFP fusion protein was almost exclusively expressed in cholinergic cells in the hypoglossal motoneuron pool. Additionally, 94.1% of ChAT expressing cells in the hypoglossal motoneuron pool also expressed EYFP (370 of 393 cells identified from three slices each from three ChAT-ChR2(H134R)-EYFP mice). Together, these results indicated that, as identified by immunohistochemistry, almost all of the cells that expressed the opsin were identified as cholinergic, but of the cells that were identified as cholinergic not all expressed the opsin.

Analyses of colocalization of the ChR2(H134R)-EYFP fusion protein with c-fos expression in the hypoglossal motoneuron pool is included in the *Supplemental Information* and Fig. S1. Those data identified that c-fos: (i) was not only expressed in the hypoglossal motoneuron pool in the ChR2(H134R)-EYFP mice, i.e., other neuronal groups also expressed c-fos; (ii) was not a specific marker to the hypoglossal motoneuron pool that was targeted by optogenetic stimulation as it was expressed elsewhere; and (iii) was also present in the control C57BL/6 mice whether they were subject to optical stimulation or not, i.e., it was not a specific marker of optical stimulation in this case.

### Study 1: Experiments under isoflurane anesthesia

**Figure 2:**
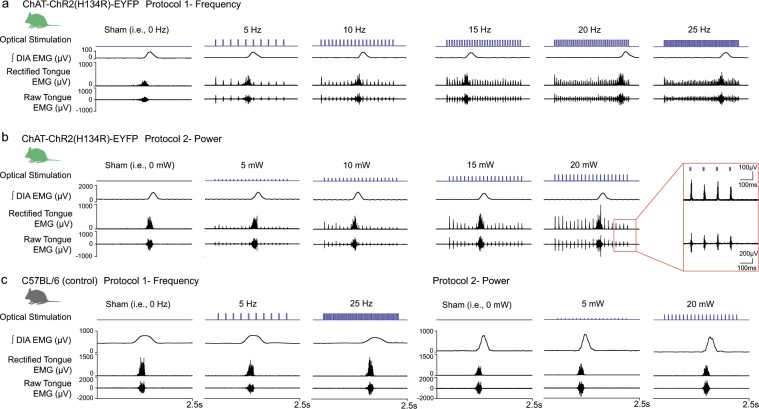
Tongue motor responses elicited by photostimulation under isoflurane anesthesia. Examples of the tongue motor responses in ChAT-ChR2(H134R)-EYFP mice elicited by photostimulation in (**a**) *Protocol 1 – Frequency* (5, 10, 15, 20 and 25 Hz) and (**b**) *Protocol 2 – Power* (5, 10, 15 and 20 mW). Each stimulus train lasts 2 s and spans endogenous-respiratory-related activation of the tongue and diaphragm muscles muscle. The raw and rectified tongue electromyogram (EMG) is shown, with the latter being used for measurements and analyses (see Methods). Note: (i) transient and discrete tongue motor activations elicited by each photostimulation protocol with a return to baseline between each pulse (unless the stimulation coincided with endogenous-respiratory-related activity); and (ii) the magnitude of the elicited tongue motor responses increased with increasing power of stimulation but not with increasing frequency. (**c**) No responses in the wild-type mice lacking the opsin during both the frequency or power photostimulation protocols (examples shown for 5 and 25 Hz, and 5 and 20 mW respectively).

Figure 2 shows examples of the tongue motor responses elicited by photostimulation in *Protocol 1 – Frequency* (5, 10, 15, 20 and 25 Hz, Fig. 2a) and *Protocol 2 – Power* (5, 10, 15 and 20 mW, Fig. 2b). Brief and distinct motor activations were elicited in response to *each* individual photo-pulse at *each* applied frequency and power across the range, with a return to baseline activity between each pulse. Also note that the magnitude of the elicited tongue motor responses was not consistently affected across the range of applied frequencies (*Protocol 1*, Fig. 2a), whereas increasing the power of stimulation did increase the magnitude of the motor responses (*Protocol 2*, Fig. 2b). In these anesthetized mice average breathing rates during the isoflurane-anesthesia and in the presence of hyperoxia (see Methods) were typically 20–25 breaths per minute, as observed in other studies^23,24^. We also note that the dorsal motor nucleus of the vagus is located just dorsal to the hypoglossal motoneuron pool^25^, and as such would be expected to be also activated by the optical stimuli. However, that motor nucleus does not directly innervate the tongue musculature^26,27^.

No tongue motor responses were observed in the wild-type mice lacking the opsin during either the frequency or power photostimulation protocols (Fig. 2c). This latter observation also indicates that the aforementioned responses in the ChAT-ChR2(H134R)-EYFP mice were mediated by the effects of the photostimulations on the opsins *per se* and not due to other non-specific effects (e.g., potential heat or other unknown consequences of the optical stimulation).

**Figure 3:**
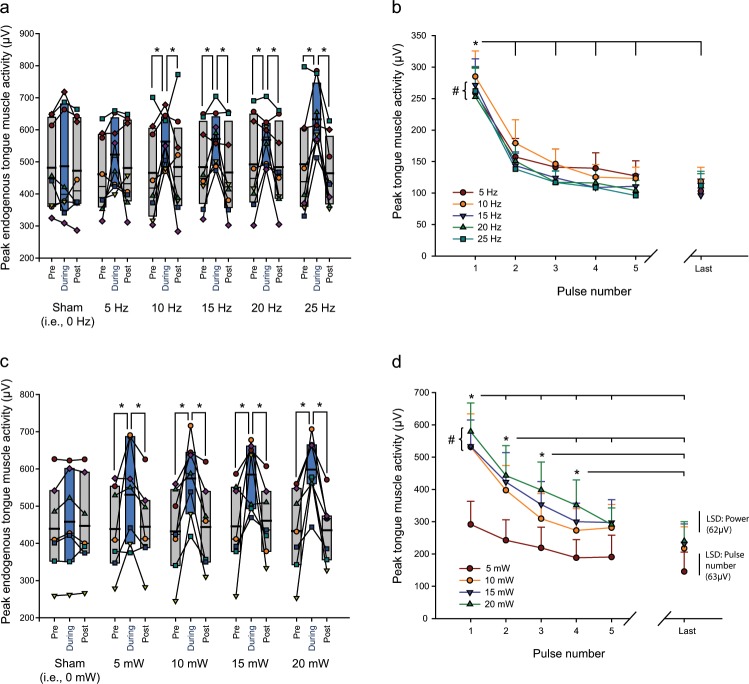
Group data for tongue motor responses elicited by photostimulation under isoflurane anesthesia. Data are shown for ChAT-ChR2(H134R)-EYFP mice in both *Protocol 1 – Frequency* (**a**,**b**, n = 8) and *Protocol 2 – Power* (**c**,**d**, n = 7). (**a**,**c**) Box and whisker plots showing the individual and group data (i.e., median, mean (thicker line), 25^th^ and 75^th^ percentiles) for tongue muscle activities. These data illustrate the effect of photostimulation on the peak amplitude of the endogenous rhythmic respiratory-related tongue EMG activity for the breath *during* stimulation compared to the tongue activity on the breath immediately before and after (i.e., *pre* and *post*) stimulation. Note that the peak amplitude of the endogenous rhythmic respiratory-related tongue activity was significantly increased (indicated by the symbol ‘*’) when the photostimulation occurred during the breath compared to pre and post, with this effect emerging at the higher frequencies (**a**) and higher powers (**c**). Each animal is represented by a different symbol. (**b**,**d**) Group mean (+SEM) data showing effect of stimulation frequency (**b**) or power (**d**) on peak elicited tongue muscle activity as a function of applied pulse number in the stimulus train (in the absence of any coincident endogenous respiratory-related activation). The symbols ‘#’ indicate a significant difference between the indicated frequencies (i.e., 10 Hz > 15, 20 and 25 Hz) or power (i.e., 5 mW < 10, 15 and 20 mW) from post-hoc tests irrespective of pulse number. The symbol ‘*’ indicate significant differences in the magnitude of responses between the indicated pulse numbers irrespective of stimulation frequency or power. d: The calculated magnitude of Fisher’s Least Significant Difference (LSD, P < 0.05) are also shown for (i) the different powers of stimulation (within a given pulse number), and (ii) the different pulse numbers in the stimulus train (within a given power). See text for further details.

Figure 3 shows individual and group data for the tongue motor responses in ChAT-ChR2(H134R)-EYFP mice elicited by photostimulation in *Protocol 1 – Frequency* (Fig. 3a,b) and *Protocol 2 – Power* (Fig. 3c,d). Average data are also shown for each of the first five and last pulse of each stimulus train (i.e., Peak EMG_1_ to Peak EMG_5_ and Peak EMG_Last_ applied at each frequency or power, Fig. 3b,d).

#### Photostimulation protocol 1 – frequency

*(i) Effect of photostimulation on endogenous respiratory-related motor activity across the range of frequencies:* There was a statistically significant effect of the presence of photostimulation on the peak amplitude of the endogenous rhythmic respiratory-related tongue muscle activity (F_2,14_ = 11.93, P < 0.001, 2-way ANOVA-RM) that depended on the frequency of photostimulation (F_10,70_ = 2.99, P = 0.003, Fig. 3a). Post-hoc analyses showed that the peak amplitude of the endogenous rhythmic respiratory-related tongue EMG activity was significantly increased when the photostimulation occurred during the breath (Peak EMG_During_) compared to the breath before or after (Peak EMG_Pre_ and Peak EMG_Post_) at stimulation frequencies of 10, 15, 20 and 25 Hz (all t_7_ > 2.63, all P < 0.023, Fig. 3a) but not sham (i.e., 0 Hz) or 5 Hz (both t_7_ < 2.11, both P > 0.114, Fig. 3a). There was no lingering effect of the photostimulation on tongue EMG activity after the breath it was applied, with Peak EMG_Post_ being statistically indistinguishable from Peak EMG_Pre_ across the full range of frequencies (i.e., 0–25 Hz, all t_7_ < 0.97, all P > 0.336, Fig. 3a).

This activating effect of optical stimulation on the peak amplitude of the endogenous rhythmic respiratory-related tongue EMG activity was specific to the tongue, as there was no effect on the amplitude of diaphragm activity pre-, during, or post-stimulation (F_2,14_ = 1.00, P = 0.392, 2-way ANOVA-RM).

*(ii) Effect of photostimulation frequency on elicited tongue EMG activity:* There was also a statistically significant main effect of stimulation frequency on the peak elicited tongue motor activity across photostimulation pulses (F_4,28_ = 5.42, P = 0.002, 2-way ANOVA-RM, Fig. 3b), with overall responses to 10 Hz stimulation being greater than for 15, 20 and 25 Hz (all P < 0.022, post-hoc Holm-Sidak tests, see symbol ‘#’ in Fig. 3b). There was also a significant main effect of stimulation pulse number on the peak elicited tongue motor activity across the range of stimulation frequencies (F_5,35_ = 25.37, P < 0.001, 2-way ANOVA-RM, Fig. 3b), with responses elicited by the first pulse being greater than for the subsequent pulses (i.e., second through fifth, and the last pulse of the stimulus train, all P < 0.001, post-hoc Holm-Sidak tests, see symbol ‘*’ in Fig. 3b). The effect of stimulation frequency on the peak elicited tongue motor activity did not depend on pulse number, i.e., there was not a significant interaction between these two factors (F_20,140_ = 1.30, P = 0.187, 2-way ANOVA-RM).

#### Photostimulation protocol 2 – power

*(i) Effect of photostimulation on endogenous respiratory-related motor activity across the range of powers:* There was a statistically significant effect of photostimulation power on the peak amplitude of the endogenous rhythmic respiratory-related tongue muscle activity (F_8,48_ = 4.21, P < 0.001, 2-way ANOVA-RM, Fig. 3c). Post-hoc analyses showed that the peak amplitude of the endogenous rhythmic respiratory-related tongue EMG activity was significantly increased when the photostimulation occurred during the breath (Peak EMG_During_) compared to the breath before or after (Peak EMG_Pre_ and Peak EMG_Post_) across the range of stimulation powers (i.e., 5–20 mW, all t_6_ > 2.77, all P < 0.019, Fig. 3c) but not sham (i.e., 0 mW, both t_6_ < 0.61, both P > 0.908, Fig. 3c). There was also no lingering effect of the photostimulation on tongue EMG activity after the breath it was applied, with Peak EMG_Post_ being statistically indistinguishable from Peak EMG_Pre_ across the range of stimulation powers (i.e., sham, and 5–20 mW, all t_6_ < 0.50, all P > 0.624, Fig. 3c).

This activating effect of optical stimulation at the various powers on the peak amplitude of the endogenous rhythmic respiratory-related tongue EMG activity was specific to the tongue, as there was also no effect on the amplitude of diaphragm activity (F_2,12_ = 1.19, P = 0.339, 2-way ANOVA-RM).

*(ii) Effect of individual photostimulation pulses on elicited tongue EMG activity across the range of powers:* There was a statistically significant main effect of stimulation power on peak elicited tongue motor activity (F_3,18_ = 23.49, P < 0.001, 2-way ANOVA-RM, Fig. 3d), with responses to 10, 15 and 20 mW being greater than for 5 mW (all P < 0.001, post-hoc Holm-Sidak tests, see symbol ‘#’ in Fig. 3d). There was also a significant main effect of pulse number on the peak elicited tongue EMG activity (F_5,30_ = 31.86, P < 0.001, 2-way ANOVA-RM, Fig. 3d). The responses elicited by the first pulse were significantly greater than for all the subsequent pulses (i.e., second through fifth, as well as the last pulse of the train, all P < 0.001, post-hoc Holm-Sidak tests, see symbol ‘*’ in Fig. 3d). Likewise, the responses elicited by the second pulse were greater than for the fourth, fifth and last pulses (each P < 0.003, see ‘*’ in Fig. 3d). The responses elicited by the third and fourth pulses were also greater than the response elicited by the last pulse (P < 0.001 and P = 0.046 respectively, see symbols ‘*’ and ‘*’ in Fig. 3d). Together, these changes in the magnitude of response across stimulation pulse number are likely indicative of a process of ChR2 desensitization^28–30^.

There was a significant interaction between stimulation power and pulse number (F_15,90_ = 4.18, P < 0.001, 2-way ANOVA-RM), i.e., the magnitude of the motor activating effects of the different levels of power depended on the pulse number (Fig. 3d). Post-hoc analyses identified the magnitude of Fisher’s Least Significant Difference (P < 0.05, Fig. 3d) for the magnitude of responses to: (i) the different powers of stimulation (within a given pulse number), and (ii) the different pulse numbers in the stimulus train (within a given power). In summary, these data identify that the optically-elicited tongue motor responses were larger with higher powers, and also larger toward the beginning of the stimulation train, with the differences increasing from lower to the higher powers (Fig. 3d).

### Study 2: Experiments across sleep and awake states

Figure S2 in the *Supplemental Information* shows representative baseline tongue muscle activity recorded across sleep-wake states.

**Figure 4:**
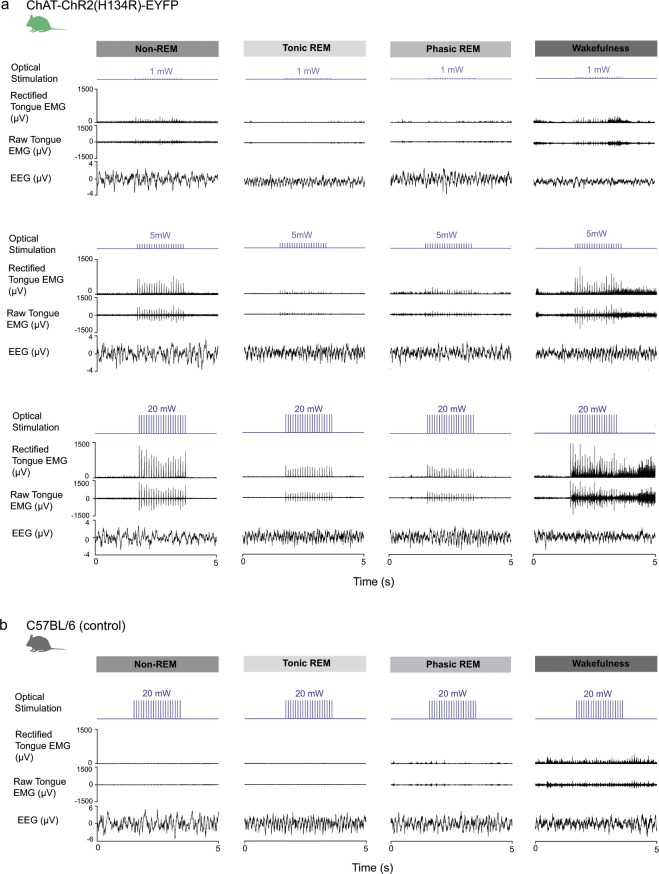
Tongue motor responses elicited by photostimulation across sleep-wake states. (**a**) Examples of the tongue motor responses in ChAT-ChR2(H134R)-EYFP mice elicited by photostimulation at different powers (1, 5 and 20 mW are shown) for non-REM and REM sleep, and wakefulness. In the phasic periods of REM sleep there is underlying fluctuating postural muscle tone that is also apparent in the tongue. Each stimulus train lasts 2 s. The raw and rectified tongue electromyograms (EMGs) are shown, with the latter being used for measurements and analyses (see Methods). Note: (i) increased tongue motor responses with increased stimulation power within a given sleep-wake state; and (ii) reduced responses in REM sleep. See text for further details. (**b**) No tongue motor responses are observed in response to photostimulation in the wild-type mice lacking the opsin.

*(i) Example responses to photostimulation across sleep-wake states:* Fig. 4a shows examples of the tongue motor responses in ChAT-ChR2(H134R)-EYFP mice elicited by photostimulation in sample periods of wakefulness, non-REM and REM sleep. Of note: (i) within any given sleep-wake state, the magnitude of the elicited tongue motor responses increased with increasing power of stimulation, and (ii) at any given power, the tongue motor responses elicited in REM sleep were markedly reduced compared to waking and non-REM sleep. Figure 4b shows that no tongue motor responses were observed in response to photostimulation in the wild-type mice lacking the opsin (example shown for 20 mW).

**Figure 5:**
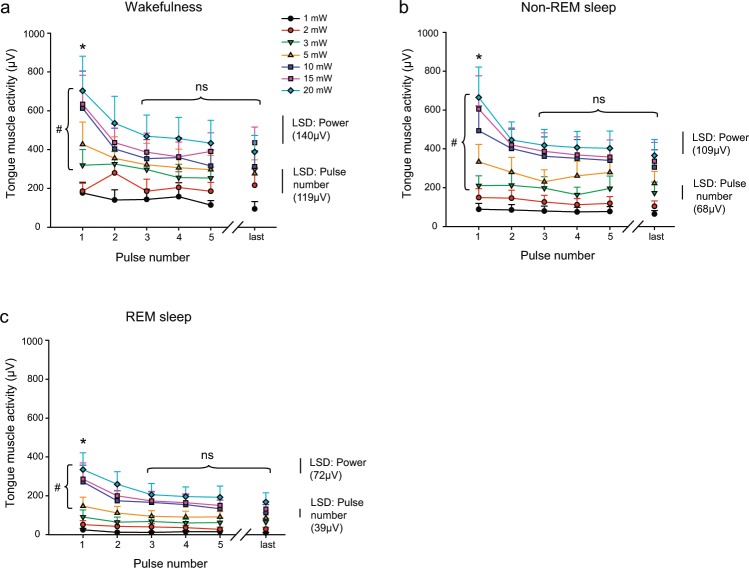
Tongue motor responses elicited by photostimulation across sleep-wake states. Group mean (+SEM) data are shown for *Protocol 3 – Power* (1, 2, 3, 5, 10, 15 and 20 mW) in ChAT-ChR2(H134R)-EYFP mice (n = 7). These data illustrate the effect of stimulation power on peak elicited tongue muscle activity as a function of applied pulse number in the stimulus train for interventions performed in wakefulness (**a**), non-REM sleep (**b**) and REM sleep (**c**). The symbols ‘#’ indicate a significant difference between the indicated powers in each sleep-wake state from post-hoc tests irrespective of pulse number. The symbol ‘*’ indicates that the responses elicited by the first pulse were significantly larger than all the subsequent pulses (i.e., second through fifth, as well as the last pulse of the train) in each of wakefulness, non-REM and REM sleep. The abbreviation ‘ns’ indicated that the responses elicited by pulse 3 onwards were statistically indistinguishable from each other. The calculated magnitude of Fisher’s Least Significant Difference (LSD, P < 0.05) are also shown for (i) the different powers of stimulation (within a given pulse number), and (ii) the different pulse numbers in the stimulus train (within a given power). Overall, the data show that for each of wakefulness, non-REM and REM sleep the motor responses were larger with higher powers, larger at the beginning of the stimulation train, and with the magnitude of the differences in response increasing from lower to the higher powers within each sleep-wake state. See text for further details.

*(ii) Group data within sleep-wake states:* Fig. 5 shows group data for the tongue motor responses in ChAT-ChR2(H134R)-EYFP mice in wakefulness, non-REM sleep and REM sleep elicited by photostimulation. Average data are shown for each of the first five pulses and the last pulse of each stimulus train (i.e., Peak EMG_1_ to Peak EMG_5_ and Peak EMG_Last_) applied at each power. Overall, note that tongue motor responses elicited by optical stimulation in the ChAT-ChR2(H134R)-EYFP mice were (i) reduced in REM sleep compared to non-REM sleep and waking; (ii) increased with increased power of stimulation across all states; and (iii) showed evidence of desensitization with responses to the first pulse within a stimulus train being larger at higher powers compared to the responses to later pulses in the same stimulus train (e.g., pulses 3–5 and last).

For each of wakefulness, non-REM and REM sleep there was a significant main effect of stimulation power on peak elicited tongue motor activity across photostimulation pulses (each P < 0.001, 2-way ANOVA-RM). In wakefulness and non-REM sleep, powers of 3 mW and above were sufficient to elicit significant responses compared to 1 mW (each P < 0.045, post-hoc Holm-Sidak tests, see symbol ‘#’ in Fig. 5a,b). In REM sleep, powers of 5 mW and above were required to elicit significant responses compared to 1 mW (each P < 0.010, post-hoc Holm-Sidak tests, see symbol ‘#’ in Fig. 5c).

For each of wakefulness, non-REM and REM sleep there was also a significant main effect of stimulation pulse number on elicited tongue motor activity (each P < 0.001, 2-way ANOVA-RM). In each of these states, the response elicited by the first pulse was significantly larger than all the subsequent pulses (i.e., second through fifth, as well as the last pulse of the train, range of P = 0.047 to <0.001, post-hoc Holm-Sidak tests, see symbol ‘*’ in Fig. 5a–c), indicating a process of ChR2 desensitization occurring after the first stimulation. In each of wakefulness, non-REM and REM sleep the responses elicited by the third pulse were statistically indistinguishable from the fourth, fifth and last pulses (each P > 0.413, see symbol ‘ns’ in Fig. 5a–c), i.e., any desensitization was complete after the second pulse, and the responses were statistically similar thereafter.

There was a significant interaction between stimulation power and pulse number in each of wakefulness, non-REM and REM sleep (each P < 0.001), i.e., the magnitude of the motor activating effects of the different levels of power depended on the pulse number. For each of wakefulness, non-REM and REM sleep, post-hoc analyses identified the magnitude of Fisher’s Least Significant Difference (P < 0.05, Fig. 5a–c) for the magnitude of responses: (i) to the different powers of stimulation (within a given pulse number), and (ii) to the different pulse numbers in the stimulus train (within a given power). In summary, these data identify that for each of wakefulness, non-REM and REM sleep the optically-elicited tongue motor responses were: (i) larger with higher powers, (ii) larger at the beginning of the stimulation train, and (iii) with the differences in response increasing from lower to the higher powers within each sleep-wake state (Fig. 5a–c).

**Figure 6:**
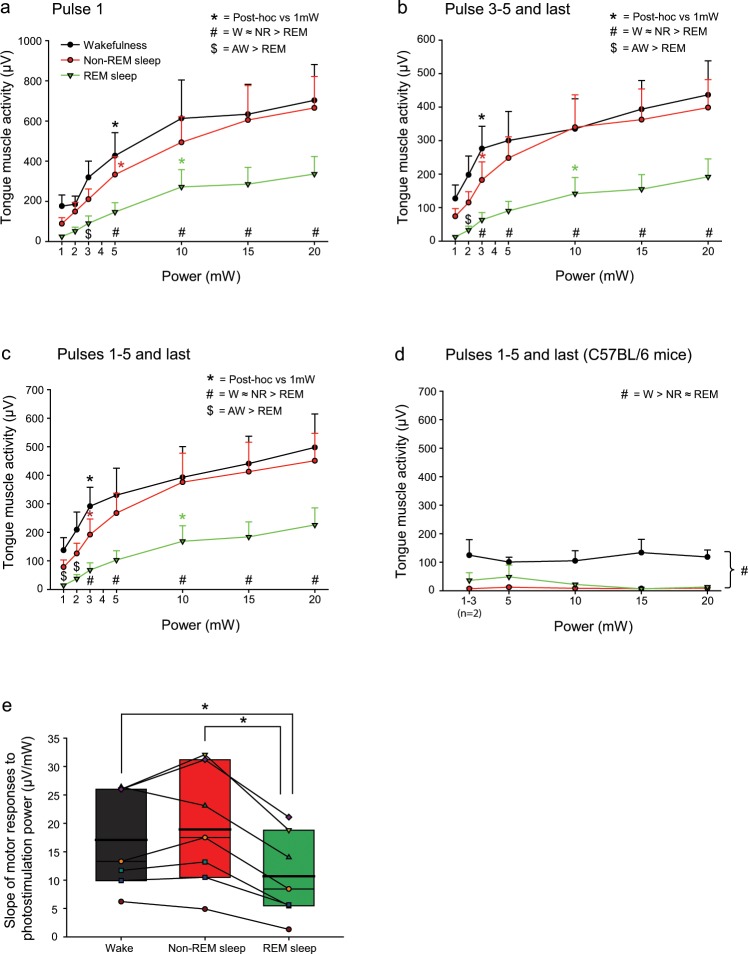
Group data for the effects of sleep-wake state on the tongue motor responses elicited by photostimulation. Group mean (+SEM) data are shown for *Protocol 3 – Power* (1, 2, 3, 5, 10, 15 and 20 mW). (**a**–**c**) These data illustrate the effect of stimulation power on peak elicited tongue muscle activity as a function of applied pulse number in the ChAT-ChR2(H134R)-EYFP mice. (**a**) responses to the first stimulation pulse (i.e., before desensitization), (**b**) responses from the third to fifth and last pulse (i.e., after desensitization) and (**c**) pooled for all pulses (i.e., irrespective of desensitization). The symbol ‘*’ indicates when the response becomes statistically significant in each sleep-wake state compared to the lowest applied power (i.e., 1 mW). Note that response thresholds are consistently lowest in REM sleep compared to non-REM sleep and wakefulness. The symbol ‘#’ indicates when responses at any given power are significantly reduced in REM sleep compared to non-REM and wakefulness. The symbol ‘$’ indicates when responses at any given power are significantly reduced in REM sleep compared to wakefulness only. (**d**) Tongue muscle activity during application of the same interventions in the wild-type mice lacking the opsins (n = 3 for all data points except for n = 2 for 1–3 mW). Note that the prevailing baseline tongue muscle activity is significantly higher in wakefulness compared to non-REM and REM sleep across the range (indicated by the symbol ‘#’) but that there is no effect of changes in power in the wild-type mice, i.e., the differences are due to ongoing sleep-wake state *per se* and not the optical interventions. (**e**) Box and whisker plots showing the individual and group data (i.e., median, mean (thicker line), 25^th^ and 75^th^ percentiles) for the slopes of the regressions of output motor responses to the magnitude of input photostimulation powers in the ChAT-ChR2(H134R)-EYFP mice. The data are derived from the output motor responses pooled for all pulses (i.e., irrespective of opsin desensitization, panel c). Individual points derive from individual mice in each sleep-wake state. The symbol ‘*’ indicates significant reductions in REM sleep compared to both non-REM sleep and wakefulness.

*(iii) Differences across sleep-wake states:* Fig. 6a–c shows overall group data for the effects of sleep-wake state on the tongue motor responses elicited by photostimulation in ChAT-ChR2(H134R)-EYFP mice. Averaged data are shown for responses before desensitization (Fig. 6a, i.e., responses to the first photostimulation pulse,) and after desensitization (Fig. 6b, i.e., responses from the third to fifth and last pulse within a stimulus train). These data are also pooled and analyzed for all pulses (Fig. 6c). As can be seen from the symbols on each figure that indicate the results of the statistical comparisons detailed below, the results and their interpretation apply to all the data, i.e., before desensitization (Fig. 6a), after desensitization (Fig. 6b) and irrespective of desensitization (Fig. 6c).

In each case (i.e., Fig. 6a–c) there was a significant interaction between stimulation power and the magnitude of elicited tongue motor activation in wakefulness, non-REM and REM sleep (each P < 0.001, 2-way ANOVA-RM). Higher powers of optical stimulation were required to elicit tongue motor activation in REM sleep. Significant tongue motor activation occurred at stimulation powers of 3–5 mW in wakefulness and non-REM sleep, and 10 mW in REM sleep (see symbol ‘*’in Fig. 6a–c, P < 0.05, post-hoc Holm-Sidak tests *vs*. lowest applied power).

Excitability of motor responses in REM sleep were also reduced across the range of applied powers. Statistical analyses identified that responses were significantly reduced in REM sleep, compared to both non-REM sleep and wakefulness, at powers of 3–5 mW through to 20 mW (see symbol ‘#’in Fig. 6a–c, P < 0.05, post-hoc post-hoc Holm-Sidak tests). At lower powers (2 and 1 mW) significant reductions in responses were also identified in REM sleep compared to those in wakefulness (see symbol ‘$’in Fig. 6a–c, P < 0.05, post-hoc Holm-Sidak tests).

Further analyses also support the above finding that the excitability and responsivity of hypoglossal motor output is significantly reduced in REM sleep compared to non-REM sleep and wakefulness. In each ChAT-ChR2(H134R)-EYFP mouse, in each sleep-wake state, regression analyses were performed on the relationship between input photostimulation powers and output motor responses. Figure 6d shows individual and group data for the slopes of these regressions and the significant effect of sleep-wake state (F_2,12_ = 25.38, P < 0.001, 1-way ANOVA-RM, Fig. 6d). Post-hoc analyses identified that the slopes of these regressions between input photostimulation powers and output motor responses in REM sleep (10.7 ± 2.8 μV/mW) were significantly lower than non-REM sleep and wakefulness (18.9 ± 3.9 and 17.1 ± 3.3 μV/mW respectively, both t_6_ > 5.26, both P < 0.001, Holm-Sidak tests). The slopes of the regressions were similar between non-REM sleep and wakefulness (t_6_ = 1.53, P = 0.153, post-hoc Holm-Sidak test).

*(iv) Reproducibility of responses:* The stimuli of 1, 2, 3, 5, 10, 15 and 20 mW were applied in random order as a block of stimuli across sleep-wake states (see Methods). Multiple blocks (each in random order) were applied across the whole study, comprising up to 100 stimuli per mouse. The magnitude of tongue motor responses for each stimulation power was not significantly different across the stimulation blocks for each of wakefulness, non-REM sleep and REM sleep (P = 0.942, 0.431 and 0.791 respectively, 2-way ANOVA-RM), i.e., the elicited motor responses were reproducible within each state.

*(v) Lack of responses in the wild-type mice:* Importantly, and as expected, in the wild-type mice there was no effect of stimulation power on the magnitude of tongue motor activity in wakefulness, non-REM sleep or REM sleep (F_3,6_ = 0.27, P = 0.843, 2-way ANOVA-RM, Fig. 6d). As expected, we also identified a statistically significant effect of sleep-wake state *per se* on tongue muscle activity (F_2,4_ = 11.93, P = 0.021, 2-way ANOVA-RM), with activity in wakefulness being significantly higher than in non-REM and REM sleep (each P < 0.035, post-hoc Holm-Sidak tests, see symbol ‘#’ in Fig. 6d). Activity in non-REM sleep and REM sleep were not significantly different from each other in these mice (P = 0.599, post-hoc Holm-Sidak test). This effect of sleep-wake state on tongue motor activity did not depend on stimulation power (F_6,12_ = 1.02, P = 0.458, 2-way ANOVA-RM) indicating that the levels of tongue muscle activity measured during stimulation in these wild-type mice lacking the opsin were the result of the prevailing sleep-wake *per se* and not due to any potential non-specific effects (e.g., heat or some other unidentified consequence of photostimulation). These data on the lack of responses in the wild-type mice are also in agreement with the data reported and shown in Figs. 4b and 2c.

Analysis of the effects on other aspects of respiratory activity are included in the *Supplemental Information*.

## Discussion

The individual tongue motor responses elicited at any given power of optical stimulation reflect the net synchronized response of the population of stimulated neurons. Distinct motor activations were elicited in response to each individual optical pulse at each applied power and frequency across the range, with a return to baseline activity between pulses (Figs. 2 and 4). The return to baseline between individual pulses occurred as only one action potential is normally elicited by a given light pulse in ChR2(H134R)-expressing neurons^30^.

Increased power of optical stimulation elicited increased tongue motor responses. Henneman’s size principle identifies that in response to a given excitatory input, smaller neurons are more excitable because input resistance is inversely related to cell size^31^. Motor unit recruitment follows this physiological pattern of recruitment sequence with optical stimulation^32^. As such, smaller neurons would be recruited preferentially with photostimulation in any given plane, with larger neurons being recruited at higher powers and contributing to the larger resulting responses. In addition, with increased powers of optical stimulation additional neurons would be excited in deeper planes, again according to Henneman’s size principle, thus also contributing to the larger responses with increased power.

It is also relevant to note that the significant activating effect of optical stimulation (Fig. 2) was specific to the tongue, as there were no significant effects on diaphragm activation pre-, during, or post-stimulation. As such, the effects on tongue muscle activity were the direct result of the optical stimulations *per se* and not due to changes in other components of the respiratory circuitry or respiratory parameters. The dorsal motor nucleus of the vagus would also be expected to be activated by the optical stimuli given its position directly above the hypoglossal motor nucleus^25^ but it does not project to the tongue musculature^26,27^. It is unknown if there were any gastrointestinal consequences of optical stimulation of the dorsal motor nucleus of the vagus but there were no obvious changes in animal behavior noted during the experiments.

In REM sleep there were significant: (i) reductions in tongue motor responses at any given power of photostimulation, (ii) elevations of the threshold power required to elicit a motor response, and (iii) decreased slopes of the regression between input photostimulation powers and output motor responses (Fig. 6). Together these REM sleep-specific outcome measures are indicative of decreased hypoglossal motor excitability and responsivity in REM sleep. To our knowledge these are the first demonstrations of hypoglossal motor excitability and responsivity in mice and are also directly relevant to the pathophysiological and phenotypic traits identified as significant in OSA^5–8^. Such a reduction in hypoglossal motor excitability and responsivity in REM sleep is interpreted, at least in part, to result from the hyperpolarization, decreased input resistance and increased rheobase as identified from intracellular recordings of hypoglossal motoneurons during the REM-like state induced by pontine carbachol in decerebrate cats^33,34^ and from one study during natural REM sleep also in cats^35^.

The advantages of applying such a photostimulation protocol to identify the physiological properties and state-specific changes in net hypoglossal motor excitability and responsivity include obviating the need for intracellular recordings and a head-restraining device to make cellular recordings in a limited number of neurons and animals; those latter requirements severely limiting such studies across states of behavior to one study of a total of 12 hypoglossal motoneurons in REM sleep^35^.

There are limitations to the present study. Figure S1 showed c-fos signal in the hypoglossal motoneuron pool and other areas of the medulla in the ChAT-ChR2(H134R)-EYFP mice. Such a widespread expression of c-fos could be taken to suggest widespread neuronal activation in the medulla in response to the photostimulation. However, Fig. S1 also showed c-fos signal in the hypoglossal motoneuron pool of control C57BL/6 mice whether or not they were subject to optical stimulation. The common feature to those three conditions in Fig. S1 is the presence of general anesthesia induced by isoflurane. A related volatile anesthetic, halothane, elicits c-fos in this region of medulla in rats, including in neurons that are not retrogradely labelled from the hypoglossal motor nucleus^36^, with the distributions overlapping those in Fig. S1. It is also noted that Fig. S1 does not show the normal and expected restricted nuclear expression of c-fos. Extra-nuclear c-fos has been observed in few other studies, e.g.^37,38^, but was not commented upon by those authors. However, we suggest that the most parsimonious explanation for the widespread including non-nuclear-restriction of the c-fos ‘signal’ in Fig. S1 (including in the control, non-opsin-containing, mice with and without photostimulation) was that this expression was not due to optical stimulation and was not a specific marker of optically-activated cells. This non-specificity occurred despite our using the same antibody as used by others e.g.^39,40^. For the following three reasons, however, we do not view this lack of specificity in the c-fos signal as a major concern for the main findings and interpretation of this paper, i.e., that variations in photostimulation input produce tunable changes in hypoglossal motor output *in-vivo* and they identify REM sleep specific suppression of net motor excitability and responsivity.

**Figure 7:**
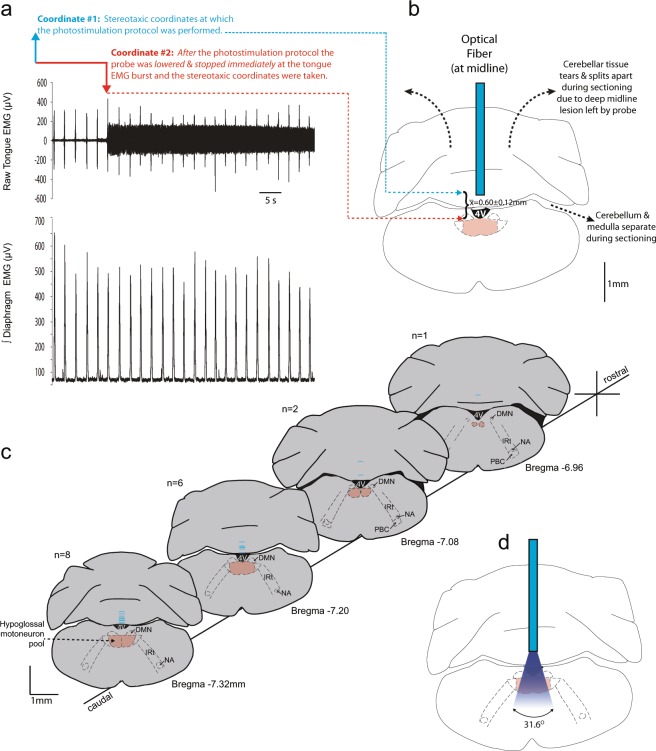
Protocol used to determine the locations of the optical probes above the medullary surface and hypoglossal motor nucleus in Study 1, with the average coordinates used for probe placements in Study 2. (**a**,**b**) Using the protocol detailed in the Methods (*Protocols and Optical Probe Placement*) the average distance between the relative location of the hypoglossal motoneuron pool and the selected photostimulation depth for subsequent data analyses was estimated as the coordinate at which the probe elicited an abrupt motor response due to physical disturbance of the tissue at the end of the experiment (Coordinate #2) minus the coordinate above at which the probe was positioned for the preceding photostimulation experiment (Coordinate #1). For the 17 mice used for all the experiments in Study 1 (i.e., ChAT-ChR2(H134R)-EYFP and C57BL/6) the average distance of the probe site above the hypoglossal motoneuron pool was estimated and calculated as 0.60 ± 0.12 mm. (**c**) Reconstructed optical probe locations on coronal sections of the mouse brain for all mice using the protocol identified in a-b above. The ventral tips of the optical probes are represented by the horizontal blue lines. (**d**) Using the procedure and equation also detailed in the Methods, the divergence angle of the optical stimulation was calculated and visualized. The estimated cone of light illumination does not include any additional spread due, for example, by random light scattering and absorption by tissue and the fourth ventricle. The diminution of power with increasing distance from the fiber tip is represented as decreased intensity of the blue light for visual purposes only. *Abbreviations:* 4V, fourth ventricle; DMN, dorsal motor nucleus of the vagus; IRt, intermediate medullary reticular region; NA, nucleus ambiguus; PBC, pre-Bötzinger complex.

Firstly, almost all of the cells that expressed the opsin were identified as cholinergic (99.7%). As such the optical stimulation will activate cholinergic neurons as has been well characterized^30^. Secondly, the opsin-containing cells are strongly present in the region of the hypoglossal motoneuron pool, as well as the dorsal motor nucleus of the vagus that does not project to the hypoglossal (Fig. 1), and using the protocol described in the Methods and illustrated in Fig. 7, the probes are placed above this site. Thirdly, the results do not support the contention that the optical stimulation was indiscriminately activating cells throughout the medulla to influence the results because there were no measurable responses to photostimulation at any power or frequency in the mice that did not contain the opsin (Figs. 2c, 4b and 6d), and the distribution of cfos in non-photostimulated mice (see right panels of Fig. S1) was similar to the other groups.

We are confident that the optical probe was appropriately positioned above the medullary surface and hypoglossal motor nucleus because of the protocol detailed in the Methods and illustrated in Fig. 7. We also calculated and visualized the divergence angle of the optical stimulation (Fig. 7d) as described in the Methods using the numerical aperture of the optical probe and the refractive index of brain tissue^41–44^. It is important to note, however, that such a clean geometric cone of illumination does not represent how additional spread could be influenced by random light scattering and absorption by tissue^41,45^ as well by fluid in the fourth ventricle (Fig. 7). In addition, although power was measured and set at the fiber tip for each photostimulation protocol (see Methods), the effect of decreasing power with increasing distance from the tip^45^ is represented only as a general phenomenon for visual purposes by the decreased intensity of the blue-light^44^ (Fig. 7d).

The cholinergic cells in Fig. 1 would be expected to be stimulated by the light pulses originating from the optical probe placed above them (Fig. 7), with the recordings of motor output made from the muscle that the cholinergic hypoglossal motoneurons innervate. Nevertheless, the estimated cone of optical divergence also identifies that other opsin-containing cholinergic neurons may also be stimulated by any light impacting them either directly or indirectly via light scattering^41,45^. This includes, for example, hypoglossal pre-motor neurons of the intermediate medullary reticular region that are cholinergic^46^. Many of the neurons in this region have increased activity during REM sleep^47^, although it is unknown if these specific REM sleep-active neurons are cholinergic and if they are also the source of the motor inhibition of the hypoglossal motor pool in REM sleep^48–50^. It is also possible that a local increase in acetylcholine release at the hypoglossal motoneuron pool via activation of cholinergic pre-motor neurons could be excitatory or inhibitory^50–53^ and modulate the direct light-induced activation of the motoneurons *per se*. Since an increase in tongue motor activity is consistently elicited by each photostimulation pulse in each behavioral state including general anesthesia, the potential for any light-induced activation of inhibitory inputs (which would also be elicited in each behavioral state) must be overwhelmed by the post-synaptic excitation mediated by direct cation influx in stimulated cholinergic neurons, including hypoglossal motoneurons following the ChR2(H134R) activation.

Based on these considerations we do not conclude that *each* motor output measured in the tongue musculature in response to *each* photostimulation pulse is *only* the result of activation of hypoglossal motoneurons. Other cholinergic neurons innervating the hypoglossal motoneuron pool, such as those arising from the intermediate medullary reticular region^46^ as well as their axons and terminals, would likely also have been stimulated. It is for this reason that we more conservatively conclude from the net measure of hypoglossal motor output as measured from the electrodes in the tongue musculature, that variations in photostimulation input produce tunable changes in hypoglossal motor output *in-vivo* and identify REM sleep specific suppression of net motor excitability and responsivity.

Two previous studies used cre-dependent viral vectors to transduce hypoglossal motoneurons of ChAT-Cre+ mice with hM3Dq receptors^21,22^. Those studies identified that systemic administration of clozapine-N-oxide led to increased tongue muscle activity that enlarged the pharyngeal airspace in anesthetized mice^21^, and caused sustained tongue muscle activation across sleep-wake states in behaving mice^22^. However, a key limitation of such ‘chemogenetic’ studies^21,22^ is the inability to impose acute, precise and direct control over hypoglossal motor activity using an intervention that can be transiently turned on and off, as well as graded in intensity, in order to interrogate properties of net motor excitability and responsivity. These properties are amenable to quantification using this optogenetic approach as identified and discussed below.

Variations in photostimulation parameters identified in the different protocols of the present study produce distinct changes in motor output. Importantly, these motor responses to variations in input power are used to quantify endogenous changes in net motor excitability across sleep-wake states, with the slope of the response across the range of applied powers indicating net responsiveness to given excitatory inputs. Hypoglossal motor excitability and responsivity are key pathophysiological traits that are relevant to OSA and have a complementary measure in humans as pharyngeal muscle responsiveness. As such, the present study identifies both an animal model and optogenetic approach for the quantification of relevant properties of hypoglossal motor activity across sleep-wake states that had not been previously identified. Such properties are not available for systematic quantification using chemogenetic approaches. Furthermore, the animal model and optogenetic protocol can be applied in future studies to evaluate pharmacological agents for their effects on hypoglossal motoneuronal excitability and responsivity in order to select agents for subsequent testing and evaluation.

In contrast to the significant effects on motor excitability and responsivity identified in the present study in REM sleep, the photostimulation input-output motor responses between wakefulness and non-REM sleep were statistically indistinguishable across the range of applied powers (Fig. 6). A key component of this similarity is likely the much smaller differences in cranial motoneuron membrane potential, input resistance and rheobase between wakefulness and non-REM sleep as identified in cats^54^. Measurements of the antidromic local field potential in the hypoglossal motor nucleus in response to electrical stimulation of the hypoglossal nerve in awake and sleeping cats, taken as a measure of the net level of excitability of hypoglossal motoneurons, also revealed no statistical differences between wakefulness and non-REM sleep but reduced responses in REM sleep^55^, with similar changes observed in rats^56^.

The decrements in motor responses elicited by successive pulses in the stimulus train can be attributed to properties of the opsin^28–30^. Photostimulations can result in reduced peak current with successive pulses^30,57^. This effect could lead to some neurons in the initially responsive population not reaching the threshold for subsequent activation, thus contributing to the decrements in responses observed in this study (Figs. 3 and 5) and other studies^29^. It is for this reason that the analyses across sleep-wake states (Fig. 6) were performed three ways to identify if the identification of reduced hypoglossal motor excitability in REM sleep was affected by opsin desensitization. As such analyses were performed of the elicited motor responses (i) for the first pulse only (i.e., no desensitization), (ii) pulses 3 through 5 and the last pulse (i.e., with desensitization), and (iii) pulses one through five and the last pulse (i.e., irrespective of desensitization). The data indicated that the conclusion of state-dependent modulation of the elicited motor responses to photostimulation was the same regardless of the analyses performed (Fig. 6).

The reduction in the elicited response with successive photostimulations is likely due to the aforementioned desensitization of the opsin^28–30^ but not muscle fatigue or progressive reductions in neurotransmitter release at the motor end plate. Discharge frequencies greater than those elicited in the present study have been recorded from hypoglossal motoneurons and genioglossus motor units in animal and human studies^9,12,15,18,19,35,58–64^ such that effects on fatigability or neurotransmitter release for short stimulus trains are unlikely. In addition, the elicited motor activations are within the physiological range observed during spontaneous breathing in the anesthetized mice (Fig. 2) and spontaneous tongue motor activations in the behaving mice (Fig. S2). Other studies have shown that stimulation of the hypoglossal motor nerve at 30 Hz can consistently drive motor output without apparent decrement^65^.

Pulse durations of 20 ms in the frequency protocol were shortened to 10 ms for the power protocol for reasons outlined in the Methods. In practice the frequency protocol could also have been done at 10 ms, but the only difference we may have expected would potentially be a reduction in the normal amount of motor response decay we saw as the pulses progressed within a stimulus train. However, each of the protocols stands by itself in revealing hypoglossal motor responses to varying frequency and power stimuli. Ultimately, the anesthetized studies were performed as a necessary pre-requisite to provide the framework to establish the optimal methods for the studies in wakefulness and sleep that were the main focus.

The range of frequencies chosen for the present study was physiological and selected based on animal and human studies where hypoglossal motoneurons and genioglossus motor units were recorded across sleep-wake states or general anesthesia^9,12,15,18,19,35,58–64^. We identified that in the absence of the endogenous inspiratory drive (i.e., when the photostimulations occurred between the spontaneously occurring breaths), a stimulation frequency of 10 Hz elicited greater tongue motor responses with each pulse compared to stimulations of 15–25 Hz (Fig. 3b). In addition, while motor responses were statistically higher at 10 Hz compared to 15–25 Hz, the elicited motor responses were not significantly different across 15–25 Hz. One interpretation of these findings is that 10 Hz stimulation drives the population of activated neurons close to their endogenous firing frequency, and/or at a firing frequency that allows sufficient time between the optical pulses for optimal recovery and re-establishment of the ionic gradients that cause the cellular activation in response to light-induced activation of the opsin. As such, higher stimulation rates, with correspondingly decreased time between successive activations, could yield decreased motor output^66,67^. To our knowledge, the effect of varying the frequency of stimulation of a population of hypoglossal motoneurons (electrical or optical) on the magnitude of elicited motor responses to each pulse of stimulation has not been reported, such that beyond the findings of the present study we do not know whether there is an optimal stimulation frequency that produces maximal motor output. In this context, stimulation frequencies of 30–40 Hz is common for therapeutic hypoglossal nerve stimulation, but a comparison in motor output between the various frequencies (all other variables being constant) is not available^68–70^.

The hypoglossal motoneuron pool innervates both the extrinsic and intrinsic muscles of the tongue. The former group comprises both the tongue protruders and retractors that are active during inspiration (predominantly) and expiration^71^. The extrinsic tongue muscles attach anteriorly to the mandible, posteriorly to the styloid process as well as the hyoid bone. This web of muscular attachments effectively creates a compliant upper airspace. Motoneurons innervating all these tongue muscles would have been activated in the present study and recorded as net motor output from the tongue electrodes. Studies in both animal models and humans have shown that co-activation of extrinsic (protruders and retractors) and intrinsic tongue muscles contribute to increased stability of the upper airway and resistance to airway closure^72–76^.

Anatomical confirmation of electrode sites in the tongue was performed and presented in our previous study with tongue muscle recordings in mice^22^. The surgeries for the current experiments were performed the same way, but anatomical collections of the tongue and sectioning were not performed in the present study. For this study we restrict our interpretations to ‘tongue muscle activity’, as in mice it is not possible to claim that we are recording from a particular individual muscle (e.g., genioglossus) or group of muscles (e.g., protruders).

In the studies in the anesthetized mice the average breathing rates typically averaged 20–25 breaths per minute, as in other studies^23,24^. This rate was utilized to identify if the presence of photostimulation increased the peak amplitude of the endogenous rhythmic respiratory-related tongue muscle activity during the stimulation (Peak EMG_During_) compared to the rhythmic breaths immediately prior to (Peak EMG_Pre_) and following (Peak EMG_Post_) the photostimulation. The peak amplitude of the endogenous rhythmic respiratory-related tongue muscle activity was increased when the photostimulation occurred during a spontaneous breath compared to the breaths before or after the photostimulation. This effect was observed with the power protocol at 5 mW stimulation and above (Fig. 3c), and the frequency protocol at 10 Hz stimulation and above (Fig. 3a). These findings can be best explained by the optical stimulation depolarizing the resting membrane potential of affected neurons of sufficient magnitude and duration such that the coincident arrival of the central respiratory drive potential increases net motor output; an effect analogous to how converging excitatory tonic (i.e., non-respiratory) and phasic inputs to a neuronal pool summate to determine net output and overall motor activity^77,78^.

In the sleeping mice at powers of 10–20 mW, post-stimulation respiratory rates were slightly lower than both before and during stimulation (Fig. S3). Respiratory rates were similar before and during stimulation. The lack of an immediate change in respiratory rate during the photostimulation period implicated an effect independent of the potential influence of light *per se* on neurons connected to the respiratory rhythm generating machinery (e.g., in the nucleus tractus solitarius). Rather, the appearance of a reduced respiratory rate only after optical stimulation of sufficient magnitude implicates an effect that may be due to secondary factors, e.g., changes in upper airway mechanics and/or reflexes resulting from tongue muscle activation.

In summary, this study provides a window on the behaving brain for the measurement and state-dependent modulation of net hypoglossal motor excitability and responsivity *in-vivo*. These characteristics are key pathophysiological and phenotypic traits related to human OSA.

## Methods

### Ethics approval

All procedures conformed to the recommendations of the Canadian Council on Animal Care, and the University of Toronto Animal Care Committee approved the protocols. Mice were exposed to a 12 hr light-dark cycle (7:00 h lights on), had access to food and water *ad libitum*, and were housed together before surgery and individually following surgery.

### Animals

Experiments were performed on 22 male (mean ± SD body weight = 26.9 ± 5.4 g, range = 18.0 to 38.7 g) ChAT-ChR2(H134R)-EYFP line 6 Bacterial Artificial Chromosome (BAC) transgenic mice. These mice are genetically modified to express channelrhodopsin-2 (ChR2) exclusively in cholinergic neurons (B6; Cg-Tg(Chat-COP4*H134R/EYFP,Slc18a3)6Gfng/J; stock# 014546, The Jackson Laboratory, Bar Harbor, Maine, USA)^30^. In this line, the ChR2 is modified to contain a gain of function substitution (H134R). The fusion of the opsin to enhanced yellow fluorescent protein (EYFP) allows for the visualization of ChR2-H134R expression by fluorescent microscopy. The ChR2(H134R)-EYFP fusion protein is under the control of the choline acetyltransferase (ChAT) promoter element. Given that ChAT is responsible for acetylcholine synthesis, the ChAT gene product is exclusively expressed in cholinergic cells. Consequently, the ChAT-ChR2(H134R)-EYFP mouse line displays high expression of the ChR2-EYFP fusion protein in cholinergic neurons^30^. Since motoneurons are (by definition) cholinergic, the hypoglossal motoneurons of ChAT-ChR2(H134R)-EYFP mice express ChR2(H134R) and are the main target of manipulation in the present studies. Line 6 was selected over line 5 as it has stronger expression of ChR2(H134R)-EYFP in brainstem motor nuclei^30^.

For these experiments the ChR2(H134R)-EYFP mice were purchased individually and there was no breeding colony, a strategy designed to reduce animal numbers. Given that the mice are generated on a C57BL/6 background, control experiments were performed on 5 male (mean ± SD body weight = 27.0 ± 2.8 g, range = 19.0 to 34.0 g) C57BL/6 mice lacking the opsin. These studies were performed in male mice to avoid the potential confounding effect of the estrus cycle.

### Study 1: Experiments under general anesthesia

Experiments were performed on 15 ChAT-ChR2(H134R)-EYFP mice (mean ± SD body weight = 23.7 ± 2.8 g, range = 18.0 to 26.0 g) and two C57BL/6 mice (mean body weight = 26.8 g, range = 19.0 to 26.8 g).

#### Animal preparation

Mice were studied under general anesthesia induced and maintained with isoflurane (1.5–2.5%) sufficient to abolish the pedal withdrawal and corneal blink reflexes. The level of isoflurane was set in each individual mouse to maintain surgical levels of anesthesia and then held constant for the remainder of the experiments within an animal. The range refers to differences between animals. Each animal serves as its own control, and changes within-animals are subject to the statistical analysis under a constant level of isoflurane.

Throughout the experiments the animals spontaneously breathed 50% oxygen (balance nitrogen) and were kept warm with a heating pad (T/Pump Model #TP650, Gaymar Industries, Inc., Orchard Park, NY, USA). With the mice positioned supine, bipolar stainless-steel electrodes (AS636, Cooner Wire, Chatsworth, CA, USA) were implanted onto the right side of the diaphragm as it meets the abdominal wall to record diaphragm muscle activity. Two stainless steel needle electrodes (Grass Technologies - now Natus Medical, Middleton, WI, USA) were also inserted into the tongue musculature via a per-oral approach to record tongue muscle activity. The mice were then positioned in a stereotaxic frame (Kopf model 940, Tujunga, CA, USA) with the snout secured in an anesthetic mask (Kopf model 923,Tujunga, CA, USA).

Using a dorsal approach, a mono fiber-optic probe (200/245-0.37, Doric Lenses, Quebec City, QC, Canada) was inserted into the brain at the midline (i.e., at a medial-lateral coordinate of 0 mm) and 7.23 ± 0.03 mm (mean ± SEM) posterior to bregma. The fiber-optic probe was flat at the tip with an outer diameter of 245 µm and a numerical aperture of 0.37. The optical probe was connected to the laser head and power supply (LRS-0473-GFM-00050-03, Laserglow Technologies, Toronto, ON, Canada) via an optic patch cord with a numerical aperture of 0.22 and core diameter of 50 µm (50/125/900-0.22, Doric Lenses, Quebec City, QC, Canada). The larger numerical aperture and core size of the fiber optic cannula versus the optic patch cord prevented loss of light at the connection point^79^. The laser was set to deliver light at a wavelength of 473 nm to evoke photocurrents from the blue light-sensitive ChR2^57^. The power of the light at the tip of the optical fiber was measured using a handheld digital power meter (PM100D, ThorLabs, NJ, USA) and photodiode power sensor (S130C, ThorLabs, NJ, USA).

#### Recordings

The EMG signals were amplified and band-pass filtered between 100 and 1000 Hz (Super-Z head-stage amplifiers and BMA-400 amplifiers/filters, CWE Inc., Ardmore, PA, USA). The signals were digitized at a sampling rate of 2000 Hz using a data acquisition system (CED 1401 and Spike version 6 software, Cambridge Electronic Design Ltd., Cambridge, UK).

#### Protocols and optical probe placement

For these experiments the aim was to *target* the fiber-optic probe to be ~0.6 mm above the hypoglossal motoneuron pool at the midline. However, the distance of the fiber-optic probe above the hypoglossal motoneuron pool at the time of stimulation during an experiment was unknown. For this reason, a protocol needed to be established that allowed the relative location of the optical fiber above the hypoglossal motoneuron pool to be estimated after completion of an experiment in these anesthetized animals. This protocol and procedure are as follows. The full stimulation protocol in the anesthetized mice was applied at 1 or 2 dorsal-ventral coordinates. The fiber-optic probe was first positioned at 4.85 ± 0.04 mm ventral to bregma. At this site one of the two protocols (Protocol 1 – Frequency or Protocol 2 – Power) was performed in separate groups of mice. When complete, the optical fiber was lowered again to 5.16 ± 0.03 mm ventral to bregma and the same photostimulation protocol was again performed. Once complete, the optical fiber was lowered again until the hypoglossal motoneuron pool was reached and physically disturbed as evidenced by a sudden and pronounced increase in tongue motor activity (Fig. 7) that typically lasted less than 5 min and in the absence of any light stimulation. Such an increase in tongue motor activity in response to physical disturbance also occurs when microdialysis probes are lowered to the hypoglossal motoneuron pool for microperfusion^80^. However, a notable and purposeful difference in the case of the procedures for the current experiments is that as soon as the motor pool responded to physical disturbance (likely through pressure on the membrane at the medullary surface) then *no* further ventral lowering of the probe was performed. As such no lesions in the medullary tissue were therefore observed (or expected) in any of the sections analyzed from each of the experiments in each mouse. In contrast, in microdialysis experiments the microdialysis probes are lowered an additional 0.2–0.5 mm with the aim of physically penetrating the motor pool in order that the dialysis membrane spans the motor nucleus for delivery of selected drug agents via microperfusion, and as such the lesions left by the microdialysis probes are clearly observed^80^. In the present study the aim was to not damage the hypoglossal motoneuron pool and medullary tissue in order to preserve it for subsequent histology and immunohistochemistry.

Based on the above protocol and illustrated in Fig. 7, the average distance between the relative location of the hypoglossal motoneuron pool and the selected photostimulation depth for subsequent data analyses was assessed and calculated to be 0.60 ± 0.12 mm (mean ± SEM). These values were calculated as the coordinate at which the probe elicited a motor response due to physical disturbance minus the coordinate at which the probe was positioned for the photostimulation protocol (Fig. 7). A target distance of 0.6 mm between the fiber and the motoneuron pool was considered optimal as it is expected to expose the opsin to sufficient light intensity (>10 mW/mm^2^) to maximally open the channel with 20 mW power from the fiber tip, based on the Stanford Predicted Irradiance Model in Mammalian Brain Tissue^44,57^.

Due to optical fiber being aimed above the medulla and hypoglossal motoneuron pool, at the midline, then tip of the probes were necessarily situated above the medulla surface and in the cerebellum^25^. As such anatomical images of the actual probe location could not be obtained because free-floating cerebellar tissue became torn during the cutting and solution changes required for histology thus obscuring probe locations, and because the probe location itself deep within the cerebellum caused the tissue to split apart due to the long midline lesion created (see Fig. 7). Nevertheless, from the experimentally-calculated probe locations the theoretical cone of optical stimulation could also be calculated and visualized (Fig. 7d).

The predicted divergence angle (Θ) of the cone of light during optical stimulation was calculated using the following equation: Θ = sin^−1^ (NA/*n*)^41,44^; where NA is the numerical aperture of the fiber tip (0.37) and n is the refractive index of the medium^41^ which for brain tissue was taken as 1.36^42–44^. This calculated angle (15.8 °) is considered the half-divergence angle by Aravanis *et al*.^44^, and thus the cone of light during optical stimulation is plotted as 31.6 ° in Fig. 7d. The reduction in optical power density with increasing distance from the fiber tip is illustrated by diminution of the color intensity^45^ for visual representation purposes only.

*Photostimulation protocol 1 – frequency*: 5, 10, 15, 20 and 25 Hz stimuli were applied in random order in a block of stimuli, for a total of seven blocks with the order of stimulations randomized within a block (n = 8 mice). Individual optical pulses were of 20 ms duration with the total duration of the stimulus train lasting 2 s. The power of each stimulation was set at 20 mW as measured from the fiber tip. Each stimulus train was separated by at least 15 s, allowing for near-full recovery from potential desensitization^28^. The specific range of frequencies was selected based on the endogenous tonic and respiratory-related inputs and output activities of hypoglossal motoneurons and genioglossus motor units, as recorded under general anesthesia, wakefulness and sleep across animal and human studies^9,12,15,18,19,35,58–64^.

*Photostimulation protocol 2 – power*: 5, 10, 15, and 20 mW stimuli (as measured from the fiber tip) were applied in random order in a block of stimuli, for a total of seven blocks with the order of stimulations randomized within a block (n = 7 mice). Individual optical pulses were of 10 ms duration with the total duration of the stimulus train lasting 2 s with a frequency of 10 Hz throughout. As in Protocol 1, each stimulus train was separated by at least 15 s.

With ChR2-H134R, the peak current response in achieved in less than 10 ms^57^. Regardless of the duration of the light pulse, however, only one action potential fires in response to a given pulse due to a depolarization block, such that as long as the pulse duration is long enough to achieve the peak current response, the current response would be the same regardless of the pulse duration^30^. 20 ms in the frequency protocol was shortened to 10 ms for the power protocol to allow more time for recovery from any potential desensitization should it occur between each pulse within a stimulus train.

### Study 2: Experiments across sleep and awake states

In contrast to varying the frequency of optical stimulation, motor output was significantly affected by the power of optical stimulation in a fashion akin to a stimulus-response relationship (see Results). As such a modified power photostimulation protocol (adapted from Study 1) was performed in the second set of experiments in behaving mice to test the hypothesis that there is sleep-wake state-dependent modulation of the net endogenous hypoglossal motor excitability and responsivity *in-vivo*.

For the studies in the awake and sleeping mice we added lower powers to the protocol (1, 2 and 3 mW) in addition to the 5 mW also applied in the anesthetized studies. This was done, *a-priori*, in order to be able to detect potential differences in the threshold power required to elicit a motor response between the sleep-wake states if this occurred.

Studies were performed in seven ChAT-ChR2(H134R)-EYFP mice (mean ± SD body weight = 32.6 ± 4.8 g, range = 27.8 to 38.7 g) and three C57BL/6 mice lacking the opsin (29.7 ± 4.5 g, range = 25.0 to 34.0 g).

#### Anesthesia and surgical procedures for chronic implantations

General anesthesia was induced by inhaled isoflurane (5%) with the mice in an induction chamber, and anesthesia was then maintained via a mask placed over the snout (1.5–2.5% isoflurane). Oxygen was administered to the inspired air (50% oxygen, balance air) throughout surgery. The mice were also given buprenorphine (0.5 mg/kg, subcutaneous) and meloxicam (2 mg/kg, subcutaneous) for analgesia, and dexamethasone (1 mg/kg, subcutaneous) to reduce potential brain inflammation. Effective anesthesia was judged by abolition of the pedal withdrawal and corneal blink reflexes. During surgery, body temperature was maintained with a water pump and heating pad (T/Pump-Heat Therapy System, Gaymar, Orchard Park, NY, USA).

The mice were implanted with electrodes to chronically record the electroencephalogram (EEG) and neck (trapezius) EMG electrodes for the determination of sleep–wake states, and with tongue and diaphragm electrodes for respiratory muscle recordings^22^. An electrode plug containing the EEG and EMG electrodes was custom-made before each surgery in each mouse. Each electrode was soldered onto individual pins on a female plug (FTSH-105-03-L-D, Samtec, New Albany, IN, USA) that contained the four pairs of electrode leads and the common reference.

The leads of the diaphragm and tongue muscle recording electrodes (AS632; Cooner Wire, Chatsworth, CA, USA) were tunneled subcutaneously from a cranial incision to the site of implantation. The ends of the diaphragm and neck wires were knotted into loops that were each sutured directly onto the respective muscles using non-absorbable coated silk sutures (6-0 SP-5697G, P-10, Covidien, Dublin, Ireland). The diaphragm electrodes were sutured onto the costal diaphragm through an abdominal incision. The tongue EMG electrodes consisted of a knotted end of ~1 mm diameter that was buried into a pocket in the tongue muscle that was exposed by blunt dissection along the submentum^22^. The electrode pocket was then closed using a non-absorbable coated-silk suture and the electrode lead was knotted to the suture to keep the electrode tip within the pocket. To facilitate adequate electrode placements during surgery, both the tongue and diaphragm signals were monitored on loudspeaker (AM8 Audio Amplifier, Grass) to document respiratory-related activity. All the skin incisions were then closed using absorbable sutures (4-0 GL-881, CV-15, Covidien).

The mouse was then secured in a stereotaxic apparatus for implantation of the EEG electrodes and optical fiber. The three skull electrodes (i.e., bipolar EEG electrodes plus a common reference) consisted of insulated stainless-steel wire (AS632; Cooner Wire, Chatsworth, CA, USA) attached to stainless steel screws (00-90xl/8 #303SS, J. I. Morris Co., MA, USA). The EEG electrodes were positioned in the left-frontal and right parietal skull bones, with the reference electrode positioned in the right frontal skull bone. One anchor screw was positioned in the left parietal skull bone^22,81^. Using a dorsal approach, a mono fiber-optic cannula (200/245-0.37, Doric Lenses, Quebec City, QC, Canada), subsequently referred to as the optic fiber, was inserted into the brain at the midline (i.e., at a medial-lateral coordinate of 0 mm), and 7.26 ± 0.03 mm (mean ± SEM) posterior to bregma and at a depth of 4.8 mm ventral to bregma. The optic fiber and electrode plug were secured to the skull using dental cement. The exposed end of the optical fiber was protected with a dust cap (CAP_Ferrule_1.25, Doric Lenses, Quebec City, QC, Canada). The cranial incision was closed around the electrode plug and optical fiber using absorbable sutures.

#### Habituation

The mice were habituated to the recording environment two weeks after surgery. At least 16–18 hrs prior to testing the mice were briefly anesthetized with isoflurane in an induction chamber (induced at 5% for ~30 s and maintained at 1.5 to 2.5% for typically 5–10 min). This procedure facilitated the connection of the electrode plug to the recording cable (NMUF 8/30-4046SJ, Cooner Wire, Chatsworth, CA, USA) and the optic fiber to the optic patch cord respectively, and also minimized the risk of any sudden animal movements dislodging the head piece during connections. During this connection procedure the dust cap protecting the optical fiber was removed, and the exposed tip of the optical fiber was cleaned with 10% alcohol.

The recording environment consisted of a large open-topped bowl (Rodent Bowl, MD-1514, BAS) housed within an electrically shielded and soundproofed cubicle (EPC-010, BRS/LVE). The animals were free from any disturbance, and were supplied with fresh bedding, food, and water. A video camera inside the cubicle allowed for continuous monitoring.

#### Recordings and protocol

The EEG and EMG signals were amplified, filtered and digitized as described above for the studies in anesthetized mice. The experiments across natural sleep-wake states began at 0830–0930 hrs and were performed during the day when the mice normally sleep. Because the mice were connected to the recording cable and optical fiber the day before the studies, they were undisturbed on the day of the experiments and slept freely.

*Photostimulation protocol 3 – power*: The photostimulation protocol used in the sleeping mice was modified from *Protocol 2* described for the anesthetized mice. For this modified protocol, stimuli of 1, 2, 3, 5, 10, 15 and 20 mW (as measured from the fiber tip) were applied in random order as a block of stimuli across naturally occurring states of non-REM and REM sleep, and wakefulness. Individual optical pulses were of 10 ms duration with the total duration of the stimulus train lasting 2 s with a frequency of 10 Hz throughout. Each stimulus train was separated by at least 25 s in Study 2 (as opposed to 15 s in Study 1) as this is theoretically more conservative in facilitating full recovery from any potential channel desensitization^28^. This time interval refers to the absolute minimum times between any two manually applied stimulus trains across the sleep-wake states at different powers over the course of the protocol.

Up to 100 stimuli were applied across sequential blocks, with the order of photostimulation powers within each block (i.e., 1, 2, 3, 5, 10, 15 and 20 mW) being randomized. Any missing powers in any given sleep-wake state were then identified and applied at the end of the experiment as appropriate in an attempt to obtain a full data set. In one mouse the full data set was still not complete after the mouse had been removed from the animal care facility for 24 hrs. In accordance with the conditions of the institutional animal care and use protocol, that mouse was disconnected and then returned to the animal care facility and was recorded and optically stimulated on a subsequent day to complete the protocol.

#### Identification of sleep wake states

For the purpose of applying the photostimulations, the prevailing sleep-wake states were identified during the experiments using standard criteria^22,82^. Arousals were scored according to the Sleep Disorders Atlas Task Force of the American Sleep Disorders Association^83^. The photostimulations were applied after 15–30 s of stable sleep-wake episodes. After the experiments, the particular sleep-wake states in which the stimuli were applied were confirmed independently by two individuals. Any stimuli subsequently identified to have been applied during transitional states (e.g., drowsiness, arousals from sleep and transitions from non-REM to REM sleep) were not included in the analyses.

#### Data analyses

The raw tongue muscle signal was full-wave rectified and the measurements identified below were made for all the photostimulations in each mouse for each of the two protocols in Study 1 under general anesthesia, i.e., frequency (5, 10, 15, 20 and 25 Hz) and power (5, 10, 15, and 20 mW), and for the power protocol in Study 2 across sleep-wake states (1, 2, 3, 5, 10, 15 and 20 mW). All data points were measured in Spike2 using cursors and manually recorded in a spreadsheet. All values were matched to the corresponding intervention to provide a grand mean for each variable, for each intervention, in each protocol, in each mouse.

*Effect of photostimulation on endogenous respiratory-related motor activity*: The first analysis identified if the presence of photostimulation increased the peak amplitude of the endogenous rhythmic respiratory-related tongue muscle activity during the stimulation (Peak EMG_During_) compared to the rhythmic breaths immediately prior to (Peak EMG_Pre_) and following (Peak EMG_Post_) the photostimulation. This specific analysis was confined to Study 1 in the anesthetized rats where clear respiratory-modulation of tongue muscle activity was present (see Results and *Supplementary Information*), unlike in the freely behaving mice where respiratory-modulation of tongue muscle activity was not present as the muscle was tonically active, in agreement with our previous studies^22^.

*Effect of individual photostimulation pulses on elicited tongue EMG activity*: The second analysis identified the effects of individual photostimulation pulses within a pulse train on the peak elicited tongue motor activity from each pulse. For this analysis, the peak elicited tongue motor activity for the first five pulses and the last pulse within a stimulus train (designated as Peak EMG_1_, Peak EMG_2_, … Peak EMG_5_ and Peak EMG_Last_) were measured for each intervention in each of the frequency and power protocols. Photostimulation pulses that coincided with endogenous rhythmic respiratory-related tongue activation (which obscured the response to the individual pulses) were not included in this analysis.

### Histology

At the end of the experiments all mice were overdosed with 5% isoflurane for at least 20 min. Aortic perfusion was then performed immediately using 0.1 M phosphate buffered saline (PBS) followed by 4% paraformaldehyde in PBS. The brains were removed and stored in paraformaldehyde for at least 6 hrs at 4 °C, followed by 12 to 24 hrs in 30% sucrose in PBS at 4 °C to cryo-protect the tissue. Prior to sectioning, the brainstem was isolated and frozen at −20 °C for at least 10 min. The tissue was cut into 40 µm thick coronal sections using a cryostat (Leica CM1850, Wetzlar, Hesse, Germany).

For identification of ChAT and ChR2(H134R)-EYFP expressions, sections were first rinsed in 0.1 M, pH 7.4 Tris-buffered saline (TBS). Sections were then incubated in 10% horse serum and 0.4% triton in TBS for 60 min to block non-specific immuno-reactivity. ChR2(H134R)-EYFP was identified using a mouse green fluorescent protein (GFP) primary antibody (1:1000, catalogue number: MAB3580, Millipore, Etobicoke, ON, Canada) followed by a secondary antibody (Alexa488, 1:500, code number 115-547-003, Jackson ImmunoResearch, West Grove, PA, USA). ChAT was identified using a goat ChAT primary antibody (1:100, Millipore, Etobicoke, ON, Canada) followed by a secondary antibody (Alexa647, 1:500, code number 111-605-003, Jackson ImmunoResearch, West Grove, PA, USA)^84^. Sections were incubated in the primary antibodies for 2–3 days at 4 °C and in the secondary antibodies for 1 hr at room temperature.

For identification of c-fos and ChR2(H134R)-EYFP expressions, sections were first rinsed in 0.1 M PBS and incubated in 10% goat serum and 0.4% triton in PBS. The expression of c-fos was identified using a rabbit primary antibody (1:2000, catalogue number: Ab190289, Abcam, Toronto, ON, Canada) followed by a goat anti-rabbit Alexa647 secondary antibody (1:500, Jackson ImmunoResearch, West Grove, PA, USA). ChR2(H134R)-EYFP was identified using the same antibodies as above. These sections were incubated in the primary antibodies for 24 hrs at 4 °C and in the secondary antibodies for 1 hr at room temperature. The anti-c-Fos antibody (ab190289) has been used and validated^39,40^.

In each of these protocols, cell nuclei were stained by incubating the tissue in 4′, 6-diamidine-2′-phenylindole dihydrochloride (DAPI, Life Technologies, Eugene, OR, USA) for 5 min. Finally, all sections were rinsed with 0.1 M PBS and mounted on glass slides using fluoromount (Sigma-Aldrich, Oakville, ON, Canada) to seal the tissue. To visualize expression of ChR2(H134R)-EYFP, c-fos and/or ChAT, the slides were scanned with an Axioscan Z1 slidescanner running ZEN 2 computer software (Carl Zeiss Microscopy, North York, ON, Canada) in the Microscopy Imaging Laboratory at the University of Toronto. Filter settings for DAPI, Alexa488 and Alexa647 were as follows: DAPI, emission of 465 nm; Alexa488, 529 nm; Alexa647, 668 nm. Images were taken with a magnification of 20×.

### Statistical analysis

Each mouse served as its own control. The analyses performed for each statistical test are detailed in the text where appropriate. For all comparisons, differences were considered significant if the null hypothesis was rejected at P < 0.05 using a two-tailed test. Where post-hoc comparisons were performed after analysis of variance with repeated measures (ANOVA-RM), a multiple comparison procedure (Holm-Sidak tests) was then used to determine significant differences. Analyses were performed using *Sigmaplot* version 11 (Systat Software Inc., San Jose, CA, USA). Values are shown as means ± the standard error of the mean (SEM) unless otherwise indicated as standard deviation (SD).

## Supplementary information

Supplementary Information
