# Supplementary material for: Measurement and State-Dependent Modulation of Hypoglossal Motor Excitability and Responsivity In-Vivo
Source: Sci Rep. 2020 Jan 17;10:550. doi: 10.1038/s41598-019-57328-4 (PMC6969049; doi:10.1038/s41598-019-57328-4)

**Supplemental Information**

**Measurement and State-Dependent Modulation of Hypoglossal Motor Excitability and Responsivity *In-Vivo***

Jasmin A. Aggarwal^1^, Wen-Ying Liu^2,3^, Gaspard Montandon^4,5^, Hattie Liu^4^, Stuart W. Hughes^6^, Richard L. Horner^1,4^

*^1^Department of Physiology, University of Toronto, Toronto, M5S 1A8, Canada*; *^2^State Key Laboratory of Medical Neurobiology and MOE Frontiers Center for Brain Science, Institutes of Brain Science; Department of Pharmacology, School of Basic Medical Science, Fudan University, Shanghai 200032, China; ^3^Institute of Medical Science, University of Toronto, Toronto, M5S 1A8, Canada; ^4^Department of Medicine, University of Toronto, Toronto, M5S 1A8, Canada; ^5^Keenan Research Centre for Biomedical Science, St. Michael’s Hospital, Toronto, M5B 1W8, Canada; ^6^Vertex Pharmaceuticals, Milton, Abingdon, OX14 4RW, UK.*

**c-fos:**

Figure S1 shows representative images of a coronal brain section from one ChAT-ChR2(H134R)-EYFP mouse used in the experiments, and the images show colocalization of the ChR2(H134R)-EYFP fusion protein with c-fos in the hypoglossal motoneuron pool. The figure also shows expression of c-fos was not exclusive to the hypoglossal motoneuron pool. Cell counts identified that 99.8% of visualized cells that expressed ChR2(H134R)-EYFP in the hypoglossal motoneuron pool also expressed c-fos (433 of 434 cells identified from three slices each from three ChR2-ChR2(H134R)-EYFP mice). Given the timeline for c-fos to be expressed after neuronal activation, this result may be in keeping with ChR2(H134R)-EYFP-expressing neurons in the hypoglossal motoneuron pool being active at the time of the experiments ^1,2^. However, because such c-fos expression was not exclusive to the hypoglossal motoneuron pool, being present throughout the medulla (Figure S1), this marker did not appear specific to the neuronal group targeted by optogenetic stimulation. Moreover, Figure S1 also shows c-fos expression in the hypoglossal motoneuron pool and other areas of the medulla in the control C57BL/6 mice whether or not they were subject to optical stimulation. For further comments on these findings see the Discussion in the main paper.

**Tongue muscle activity across sleep-wake states:**

Figure S2 shows tongue muscle activity across sleep-wake states. Wakefulness was associated with periods of tongue muscle activation, especially during behaviors such as eating, drinking and grooming. Tongue muscle activity was minimal in both non-REM and REM sleep, except for periods of transient tongue muscle activation appearing during phasic REM sleep along with transient (albeit smaller) occasional activations of the neck muscle.

In contrast to the recordings in anesthetized mice where there was clear respiratory-related modulation of tongue muscle activity (Figure 2), there was no discernable respiratory-related tongue muscle activity in the awake and sleeping mice (Figure S2). This latter finding is in agreement with previous recordings in mice across sleep-wake states ^3^. As such in these awake and sleeping mice we could not identify if the presence of photostimulation increased the peak amplitude of the endogenous rhythmic respiratory-related tongue motor activity during the stimulation compared to the breaths immediately prior to and following the photostimulation. That clear respiratory-related activity of tongue muscle activity was recorded in the presence of general anesthesia and not in the absence of the anesthetic is supported by previous observations and explained by the observation that certain volatile anesthetics elicit strong phasic inspiratory activity in the hypoglossal nerve via alterations in the activity of specific pre-motor neurons in the pons ^4^.

**Respiratory activity in Protocol 3:**

Analysis of the effects of photostimulation on the peak amplitude of the respiratory-related tongue EMG activity could not be determined for *Protocol 3* in the sleeping mice because activity was predominantly tonic and not respiratory-related (Figure S2), in agreement with our previous study also performed in mice across sleep and awake states ^3^. Analyses of the effects of photostimulation on respiratory activity was confined to non-REM sleep as only in that state was respiratory rate reliably stable (a characteristic of the non-REM sleep state) and uninterrupted by sporadic changes associated with active waking behaviors and the inherent variabilities associated with REM sleep.

Respiratory rate was analyzed before, during and after each photostimulation at each applied power in 6 of the 7 ChAT-ChR2(H134R)-EYFP mice (one had an unusable diaphragm EMG signal at the time of recording). Respiratory rate within the stimulation period was calculated from the number of full breaths over the duration of time for those breaths. Respiratory rate pre-and post-stimulation was likewise calculated from same number of breaths over their respective durations.

Statistical analysis identified that there was a significant interaction between photostimulation power and measured respiratory rate (F_12,60_=2.22, P=0.022). Respiratory rates immediately post-stimulation at powers of 10-20mW were slightly lower than both before and during stimulation (each P<0.043, post-hoc Holm-Sidak tests, Figure S3). However, respiratory rates were similar before and during stimulation (each P>0.208, post-hoc Holm-Sidak tests). See main manuscript for discussion.

**References**

1 Sharp, F. R., Sagar, S. M., Hicks, K., Lowenstein, D. & Hisanaga, K. c-fos mRNA, Fos, and Fos-related antigen induction by hypertonic saline and stress. *J Neurosci* **11**, 2321-2331 (1991).

2 Kovacs, K. J. Measurement of immediate-early gene activation- c-fos and beyond. *Journal of neuroendocrinology* **20**, 665-672 (2008).

3 Horton, G. A. *et al.* Activation of the Hypoglossal to Tongue Musculature Motor Pathway by Remote Control. *Scientific reports* **7**, 45860 (2017).

4 Roda, F., Pio, J., Bianchi, A. L. & Gestreau, C. Effects of anesthetics on hypoglossal nerve discharge and c-Fos expression in brainstem hypoglossal premotor neurons. *J Comp Neurol* **468**, 571-586 (2004).

**Figure Legends**

**Figure S1: Expression of c-fos.** Fluorescent microscopy images illustrating the expression of ChR2(H134R)-EYFP and c-fos in coronal brain sections from ChAT-ChR2(H134R)-EYFP and wild-type (C57BL/6) mice that had been optically stimulated. Sections from wild-type (C57BL/6) mice that had not been optically stimulated are also shown. High-magnification (a) and low-magnification (b) representative images taken from a ChAT-ChR2(H134R)-EYFP mouse at approximately 7.48mm posterior to bregma are stained for DAPI, EYFP and c-fos. The colocalization of EYFP and c-fos at the hypoglossal motoneuron pool is also illustrated (‘merge’). Note c-fos expression in each case (including in mice without optical stimulation). Also note the absence of ChR2-EYFP in the wild-type mice.

**Figure S2: Tongue muscle activity across sleep-wake states.** Examples from one ChAT-ChR2(H134R)-EYFP mouse showing the raw and/or integrated EMGs of the diaphragm, tongue and neck muscles, and the electroencephalogram (EEG), during sample periods of non-REM sleep, REM sleep, and wakefulness.

**Figure S3: Effects of photostimulation on respiratory rate in the ChAT-ChR2(H134R)-EYFP mice:** Box and whisker plots showing the individual and group data (i.e., median, mean (thicker line), 25^th^ and 75^th^ percentiles) for respiratory rates calculated *during* stimulation compared to the same number of breaths calculated immediately before and after (i.e., *pre* and *post*) stimulation. Note that respiratory rates were reduced after stimulation (indicated by the symbol ‘*’) compared to before and during stimulation, with this effect emerging at the higher powers (10-20mW). Each animal is represented by a different symbol.

**Figure S1**


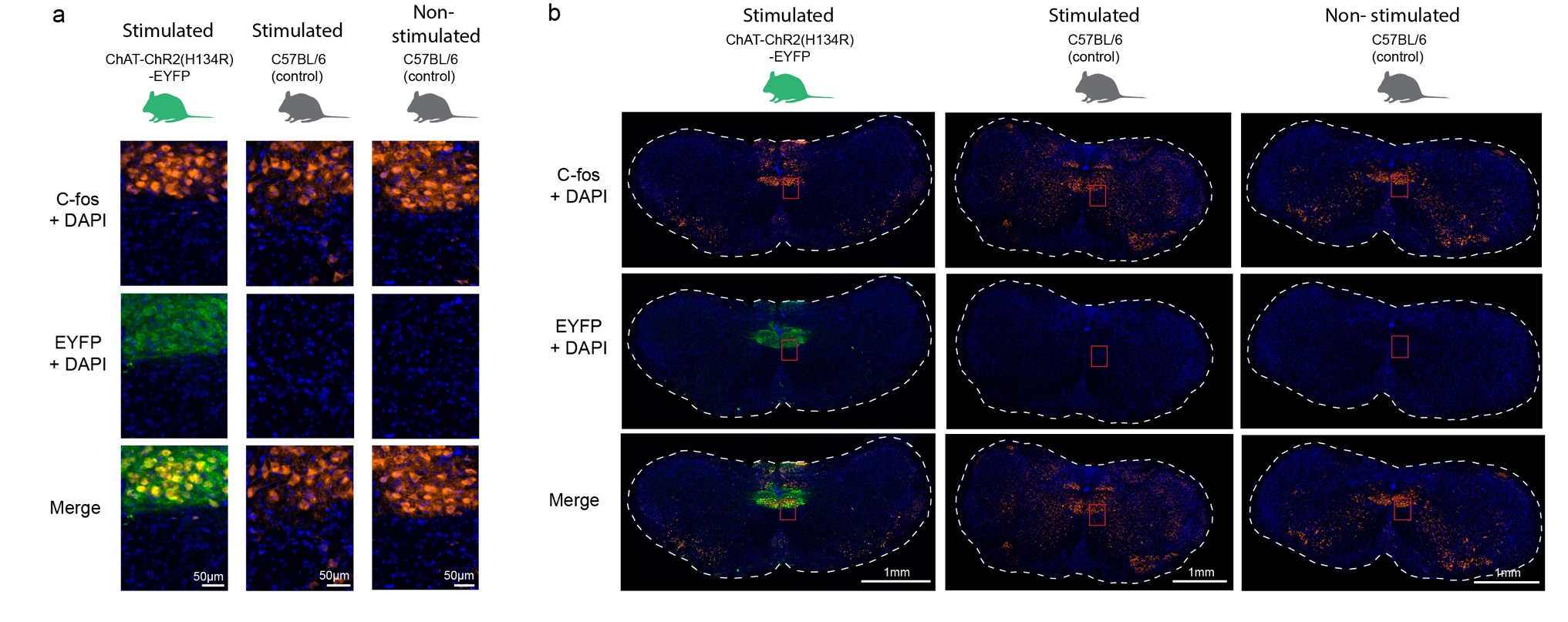


**Figure S2**

**
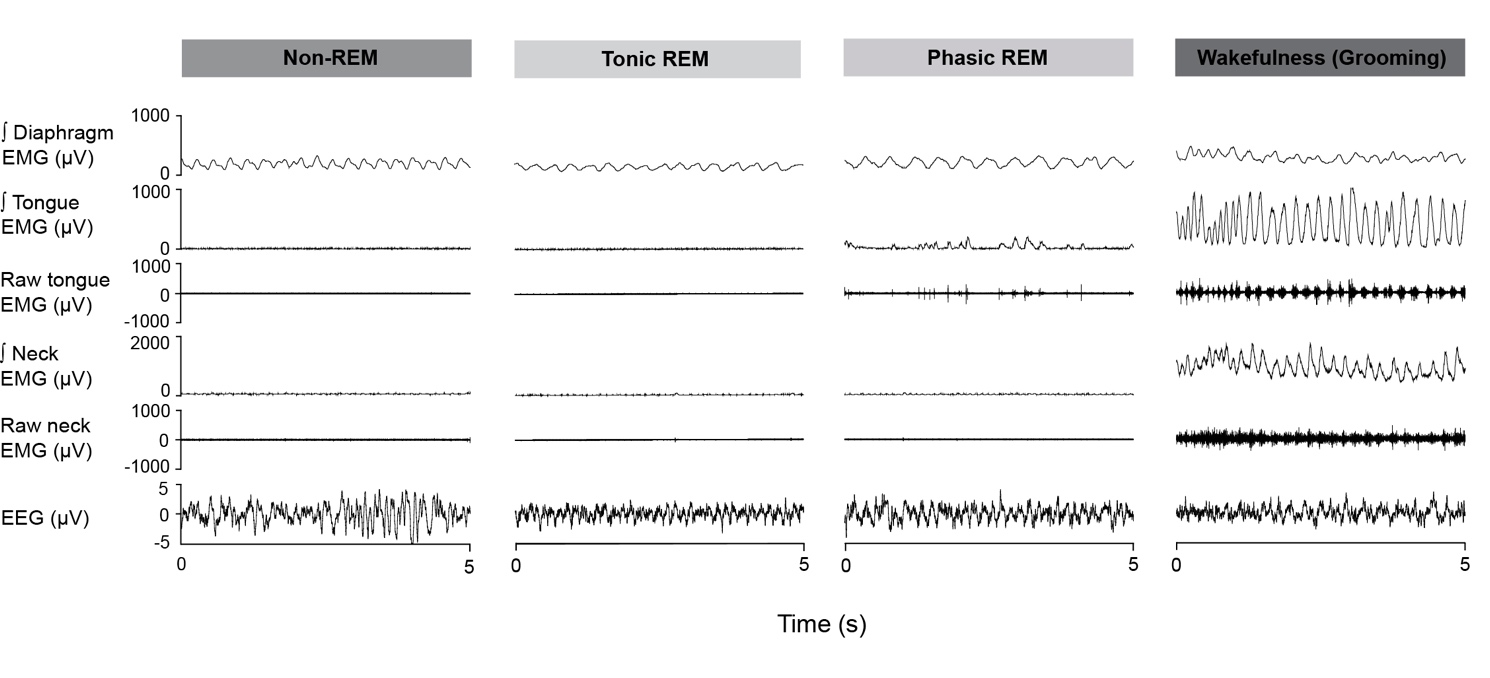
**

**Figure S3**


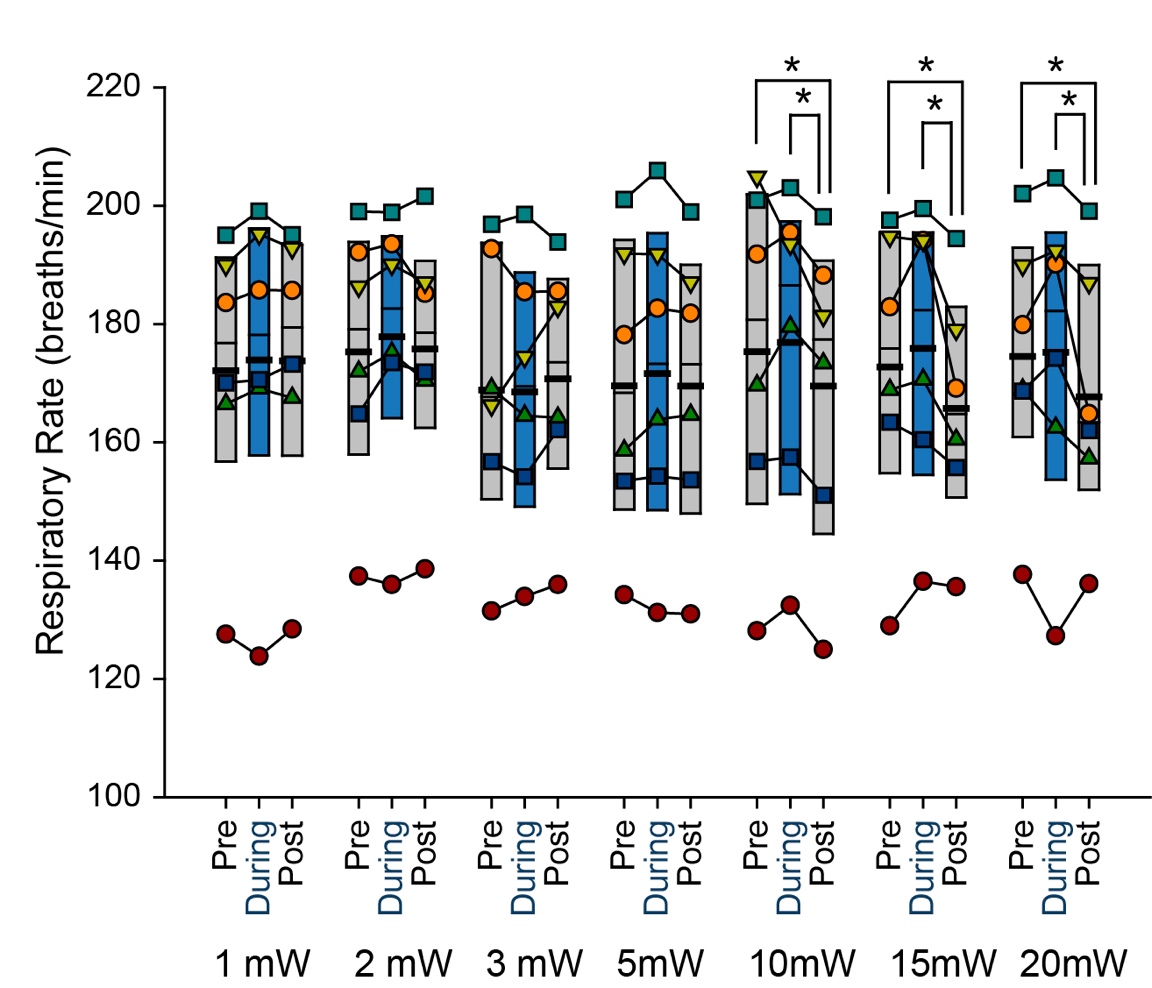

Supplement: Supplementary file 1 — Supplementary Information [file 41598_2019_57328_MOESM1_ESM.doc]
